# The Role of the Small Export Apparatus Protein, SctS, in the Activity of the Type III Secretion System

**DOI:** 10.3389/fmicb.2019.02551

**Published:** 2019-11-13

**Authors:** Irit Tseytin, Bosko Mitrovic, Nofar David, Katja Langenfeld, Raz Zarivach, Andreas Diepold, Neta Sal-Man

**Affiliations:** ^1^The Shraga Segal Department of Microbiology, Immunology and Genetics, Faculty of Health Sciences, Ben-Gurion University of the Negev, Be'er Sheva, Israel; ^2^Department of Ecophysiology, Max Planck Institute for Terrestrial Microbiology, Marburg, Germany; ^3^Department of Life Sciences and the National Institute for Biotechnology in the Negev, Ben-Gurion University of the Negev, Be'er Sheva, Israel

**Keywords:** virulence mechanism, bacterial complex, oligomerization, EscS, transmembrane domains

## Abstract

Many gram-negative pathogens utilize a protein complex, termed the type III secretion system (T3SS), to inject virulence factors from their cytoplasm directly into the host cell. An export apparatus that is formed by five putative integral membrane proteins (SctR/S/T/U/V), resides at the center of the T3SS complex. In this study, we characterized the smallest export apparatus protein, SctS, which contains two putative transmembrane domains (PTMD) that dynamically extract from the inner membrane and adopt a helix-turn-helix structure upon assembly of the T3SS. Replacement of each SctS PTMD with an alternative hydrophobic sequence resulted in abolishment of the T3SS activity, yet SctS self- and hetero-interactions as well as the overall assembly of the T3SS complex were unaffected. Our findings suggest that SctS PTMDs are not crucial for the interactions or the assembly of the T3SS base complex but rather that they are involved in adjusting the orientation of the export apparatus relative to additional T3SS sub-structures, such as the cytoplasmic- and the inner-membrane rings. This ensures the fittings between the dynamic and static components of the T3SS and supports the functionality of the T3SS complex.

## Introduction

Gram-negative bacterial pathogens, including strains of *Escherichia coli, Yersinia, Shigella, Salmonella*, and *Pseudomonas* cause serious human illness that accounts, annually, for millions of deaths worldwide (Naghavi et al., 2015; Troeger et al., 2017). These pathogens all utilize common transport nano-machines, termed the type III secretion systems (T3SSs), which translocate numerous bacterial effectors into the host cells to establish infection (Buttner, 2012; Gaytan et al., 2016; Deng et al., 2017; Wagner et al., 2018). The effectors manipulate key intracellular pathways (e.g., cytoskeletal organization, immune response, cell cycle, and metabolic processes within the host cell) that ultimately promote bacterial survival, replication, and transmission (Bhavsar et al., 2007; Buckner et al., 2011; Jayamani and Mylonakis, 2014).

The T3SS apparatus is comprised of more than 20 different proteins, most of which are found in multiple copies and are named according to the unified Sct [secretion and cellular translocation] system (Diepold and Wagner, 2014; Deng et al., 2017). The proteins are assembled into several membrane-spanning ring structures that cross the inner and outer bacterial membranes, a long needle that bridges the extracellular space, and a pore complex within the host cell membrane, to allow translocation of effector proteins (Deng et al., 2017; Wagner et al., 2018). The structural components of T3SSs of various pathogens are well-conserved and share significant similarities with components of the flagellar system (Blocker et al., 2003; Macnab, 2004; Minamino et al., 2008; Erhardt et al., 2010). For clarity, we will use the unified Sct names in the introduction section and the species-specific names of the proteins of our model organism in the results and discussion sections.

The T3SS of enteropathogenic *E. coli* (EPEC), the causative agent of pediatric diarrhea, is encoded on a 35-kb pathogenicity island found within the bacterial chromosome, termed the locus of enterocyte effacement (LEE). Among the most conserved substructures within the T3SS complex is the export apparatus, which is found at the center of the inner membrane ring, facing the cytoplasmic side. The export apparatus is composed of five proteins: SctR, SctS, SctT, SctU and SctV, with a stoichiometry of 5:4:1:1:9 in the export apparatus of the flagellar complex of *Salmonella* Typhimurium (Kuhlen et al., 2018). Null strains of single genes of the export apparatus in EPEC, in its related murine pathogen, *Citrobacter rodentium*, and in many additional T3SS-containing pathogens were found to be non-virulent, as they are defective in their ability to secrete T3SS effectors and translocators (Deng et al., 2004; Diepold et al., 2011; Yerushalmi et al., 2014; Fabiani et al., 2017; Fukumura et al., 2017; Tseytin et al., 2018b; Wagner et al., 2018). *In situ* structures of *Salmonella* Typhimurium T3SS, solved using cryo-electron tomography and sub-tomogram averaging, revealed that the export apparatus components SctR/S/T/U form a funnel-shape structure that connects to the T3SS needle on its wider end, and cross the inner-membrane on its narrow side (Hu et al., 2017). In addition, it was shown that the insertion of the export apparatus induced bending of and formation of a fenestration within the inner membrane (Hu et al., 2017). The solved structure of SctR/S/T, also called the minor T3SS export apparatus, of *Salmonella* Typhimurium flagella demonstrated that these proteins form a pseudohexameric helical structure, composed of six copies of SctT-like subunits (Kuhlen et al., 2018). The SctR_5_-SctT proteins were found to be closely associated, whereas the four SctS subunits were peripherally associated around the SctR_5_-SctT. Positioning this complex within earlier structures of flagella and T3SS basal bodies, suggested that the export apparatus complex is not embedded within the inner membrane but, rather, fits the unoccupied density at the periplasm space that was previously called “cup and socket” (Kuhlen et al., 2018). Two recent studies of the *Shigella flexneri* SctRST complex and the *Salmonella* Typhimurium T3SS needle complex observed similar localization of the complex at the periplasmic space (Hu et al., 2019; Johnson et al., 2019).

In this study, we characterized the SctS protein of EPEC, termed EscS, which is the smallest export apparatus protein (81 residues). SctS adopts a helical hairpin structure (Kuhlen et al., 2018) with two putative transmembrane domains (PTMDs) (Dietsche et al., 2016; Taylor et al., 2016). The PTMD of SctS, as well as these of SctR and SctT, are predicted to adopt transmembrane orientation at the initial assembly step of T3SS, the formation of the SctR/S/T complex within the inner-membrane, and later are extracted from the inner-membrane, by an unknown mechanism, to form the tip of the funnel-shape structure (Hu et al., 2017, 2019; Kuhlen et al., 2018; Johnson et al., 2019). To determine whether these PTMDs play a role within the T3SS or merely serve as membrane anchors or sites of unspecific hydrophobic interaction, we replaced each of them with an alternative hydrophobic sequence and examined the ability of these modified SctS versions to complement the T3SS activity of the null *sctS* strain. We found that the PTMD-exchanged versions were non-functional, thus suggesting that SctS PTMDs are critical for the activity of the T3SS. To reveal the role of these PTMDs within the complex we further investigated their involvement in SctS self- and hetero-interactions as well as their contribution to the overall assembly of the T3SS complex.

## Materials and Methods

### Bacterial Strains

Wild-type EPEC O127:H6 strain E2348/69 [streptomycin-resistant] (Iguchi et al., 2009) and EPEC null mutants (Δ*escN*, Δ*escS*, and Δ*escD*) (Gauthier et al., 2003; Tseytin et al., 2018a,b) were used to assess the T3SS and translocation activities. The *Yersinia enterocolitica (Y. enterocolitica)* strains, which bear a pYV plasmid (MRS40) (Sory et al., 1995) encoding either *egfp-yscQ* or *yscV-egfp* within the WT background or within the Δ*yscS* background, were used to assess the T3SS activity and foci formation (Diepold et al., 2010, 2011). *E. coli* BL21 (λDE3) was used for protein expression and *E. coli* DH10B, *E. coli* Top10, and DH5α were used for plasmid handling. *E. coli* FHK12 was used to assess interactions within the membranes and *E. coli* PD28 was used to examine correct orientation of the *toxR*-tmd-*malE* constructs. The *E. coli* strains (Table 1) were grown at 37°C, unless otherwise indicated, in Luria-Bertani (LB) broth (Sigma) supplemented with the appropriate antibiotics. The *Y. enterocolitica* strains were grown overnight at 28°C in brain heart infusion (BHI) medium containing nalidixic acid (35 μg/ml), and diaminopimelic acid (60 μg/ml). *Yersinia enterocolitica* day cultures were grown in BHI supplemented with nalidixic acid, diaminopimelic acid, when required, MgCl_2_ (20 mM) and glycerol (0.4%). For non-secreting conditions of *Y. enterocolitica*, 5 mM CaCl_2_ was added to the medium, whereas for secreting conditions, Ca^2+^ was chelated by addition of 5 mM EGTA. Antibiotics were used at the following concentrations: streptomycin (50 μg/mL), ampicillin (100 μg/mL), kanamycin (50 μg/mL), and chloramphenicol (30 μg/mL).

**Table 1:** Strains and plasmids used in this study.

**Strains**	**Description**	**Reference**
Wild-type EPEC	EPEC strain E2348/69, streptomycin resistant	Iguchi et al., 2009
EPEC Δ*escS*	Non-polar deletion of *escS*	Tseytin et al., 2018b
EPEC Δ*escN*	Non-polar deletion of *escN*	Gauthier et al., 2003
EPEC Δ*escD*	Non-polar deletion of *escD*	Tseytin et al., 2018a
*Y. enterocolitica* MRS40	Clinical isolate containing wild-type pYV plasmid E40 (pYVe40) Δ*blaA*	Sory et al., 1995
*Y. enterocolitica* AD4016	pYVmrs40 *egfp-yscQ*	Diepold et al., 2010
*Y. enterocolitica* AD4034	pYVmrs40 *egfp-yscQ* Δ*yscS*	Diepold et al., 2010
*Y. enterocolitica* AD4173	pYVmrs40 *yscV-egfp*	Diepold et al., 2011
*Y. enterocolitica* AD4179	pYVmrs40 *yscV-egfp* Δ*yscS*	Diepold et al., 2011
*E. coli* DH10B	For plasmid handling	Durfee et al., 2008
*E. coli* FHK12	An *E. coli* strain in which the *ctx* promoter was fused to a *lacZ* gene	Kolmar et al., 1995
*E. coli* PD28	a *malE-*deficient *E. coli* strain	Duplay et al., 1987
*E. coli* BL21 (λDE3)	For protein expression	Promega
*E. coli Top10*	For plasmid handling	Thermo Fisher
**PLASMIDS**		
pEscS_wt_-HA (pSA10)	HA C-terminal tagged EscS in pSA10	This study
pEscS_wt_-V_5_ [pET28a(+)]	V_5_ C-terminal tagged EscS in pET28a(+)	This study
pEscR-3HA (pSA10)	3HA C-terminally tagged EscR in pSA10	Tseytin et al., 2018b
pEscT-2HA (pSA10)	2HA C-terminally tagged EscT in pSA10	This study
pEscV-His (pSA10)	Penta-his C-terminally tagged EscV in pSA10	This study
pEscU_N262A_-HA (pTOPO)	2HA C-terminally tagged EscU in pTOPO – uncleavable version	Zarivach et al., 2008
pEscS-TMD1_ex_-HA (pSA10)	C-terminal tagged EscS with an 7L9A sequence instead of the original TMD1 in pSA10	This study
pEscS-TMD2_ex_-HA (pSA10)	C-terminal tagged EscS with an 7L9A sequence instead of the original TMD2 in pSA10	This study
pEscS_K54A_-HA (pSA10)	C-terminal tagged EscS with a point mutation at position 54	This study
pEscS-TMD1_ex_-V_5_ [pET28a(+)]	C-terminal tagged EscS with an 7L9A sequence instead of the original TMD1 in pET28a(+)	This study
pEscS-TMD2_ex_-V_5_ [pET28a(+)]	C-terminal tagged EscS with an 7L9A sequence instead of the original TMD2 in pET28a(+)	This study
pEscD-V_5_ (pACYC184)	V_5_ C-terminally tagged EscD in pACYC184	Tseytin et al., 2018a
pAD654	pBAD::YscS	This study
pAD655	pBAD:: YscS(P23A)	This study
pAD656	pBAD:: YscS (K54A)	This study
pAD657	pBAD:: YscS-FLAG	This study
pAD658	pBAD:: YscS (K54A)-FLAG	This study
pAD659	pBAD:: YscS (P23A)-FLAG	This study
ToxR-GpA-MBP	The GpA TMD sequence inserted between ToxR and MBP	Langosch et al., 1996
ToxR-A_16_-MBP	A sequence of 16 alanine residues inserted between ToxR and MBP	Langosch et al., 1996
ToxR-7L9A-MBP	A sequence of 7 leucine residues and 9 alanine residues inserted between ToxR and MBP	Sal-Man et al., 2005
pToxR-TMD1-MBP	The first TMD sequence of EscS inserted between ToxR and MBP	This study
pToxR-TMD2-MBP	The second TMD sequence of EscS inserted between ToxR and MBP	This study
pET28a(+)	Expression vector for His-tagging, Kan^r^	Novagen

### Construction of Plasmids Expressing EscS_wt_-HA, EscS_wt_-V_5_, EscS TMD1, and TMD2 Exchanged Labeled Both With HA and V_5_

The pSA10 plasmid was amplified using the primer pair pSA10_F/pSA10_R (Table 2). The *escS* gene was amplified from EPEC genomic DNA using the primer pairs EscS-HA_F/EscS-HA_R1 and then EscS-HA_F/EscS-HA_R2, which fused the HA tag to the coding region of *escS*. The PCR products were subjected to digestion with *Dpn*I, purified, and assembled by the Gibson assembly method (Gibson et al., 2008, 2009). A V_5_-labeled version of EscS was similarly cloned into pET28a(+); the pET28a(+) plasmid was amplified with the primer pair pET28_F/pET28_R (Table 2) and the V_5_ tag was fused to the *escS* gene using the primer pairs EscS-V_5__F/EscS-V_5__R1 and then EscS-V_5__F/EscS-V_5__R2. The PCR products were subjected to digestion with *Dpn*I, purified, and assembled by the Gibson assembly method. The resulting constructs, pEscS_wt_-HA in pSA10 and EscS_wt_-V_5_ in pET28a (+), expressed a full-length EscS protein fused to a C-terminal HA or V_5_ tag.

**Table 2:** Sequences of primers designed and used in this study.

**Construct and primer designation**	**Primer sequence**
**pEscS**_**wt**_**-HA (pSA10)**	
pSA10_F	CTGTTTCCTGTGTGAAATTGTTATCCG
pSA10_R	AATTCCCGGGGATCCGTCG
EscS-HA_F	TTTCACACAGGAAACAGatggaTACTGGATATTTTGTTCAATTATG
EscS-HA_R1	GGTAAGCGTAATCTGGAACATCGTATGGGTAGCCGTTCACCTTCGGAATC
EscS-HA_R2	GATCCCCGGGAATTTCAAGCGTAATCTGGAACATCGTATGGGTAAGCGTAATCTGG
**pEscS**_**wt**_**-V**_**5**_ **(pET28a+)**	
pET28_F	GCTGCCGCGCGGcacc
pET28_R	CTGGGATCCCCGGAATTCCC
EscS-V_5__F	GAAGGAGATATACCATGGATACTGGATATTTTG
EscS-V_5__R1	CGAGGAGAGGGTTAGGGATAGGCTTACCGCCGTTCACCTTCG
EscS-V_5__R2	CAGTCATGCTAGCCATATGTTATTACGTAGAATCGAGACCGAGGAGAGGGTTAGG
**pEscS-TMD1**_**ex**_**-HA (pSA10)**	
EscS_Fc_F	CGGCTGCAGCGGCAGCCCTGGTCCAGGCTATAACGCAG
7L9A_F	CTGTTGCTACTCTTACTCCTTGCGGCCGCAGCGGCTGCAGCGGCAGCC
7L9A_R	GGCTGCCGCTGCAGCCGCTGCGGCCGCAAGGAGTAAGAGTAGCAACAG
EscS_7L9A_F	CAAACGTTCTGGATAATATTTATCCTCCTGTTGCTACTCTTACTC
EscS_7L9A_R	CTGCGTTATAGCCTGGACCAGGGCTGCCGCTGCAGC
EscS_7L9ATM1_R	GAGGATAAATATTATCCAGAACGTTTGCAC
**pEscS-TMD2**_**ex**_**-HA (pSA10)**	
EscS_Fn_R	GTAAGAGTAGCAACAGAAAAGGCAATGTTTGATCCTGTAACTG
EscS_TM2_7L9A_F	CAAACATTGCCTTTTCTGTTGCTACTCTTACTC
EscS_TM2_7L9A_R	GATGATTGTTGTTCCCATCCAGGCTGCCGCTGCAGC
EscS_7L9AFn_F	TGGATGGGAACAACAATCATCAACTTC
**pToxR-TMD1-MBP**	
EscS_TMD1_F	CTAGCtCATTGCCTACAGTCATAGCGGCCTCTGTTATCGGTATTATTATTAGTGG
EscS_TMD1_R	GATCCCACTAATAATAATACCGATAACAGAGGCCGCTATGACTGTAGGCAATGAG
**pToxR-TMD2-MBP**	
EscS_TMD2_F	CTAGCTTGCTAAAAATAATAGCAGTGTTTGCTACGCTTGCCCTGACTTATCACGG
EscS_TMD2_R	GATCCCGTGATAAGTCAGGGCAAGCGTAGCAAACACTGCTATTATTTTTAGCAAG
**pEscV-His (pSA10)**	
EscV_F	TCACACAGGAAACAGATGAATAAACTCTTAAATATATTTAAAAAAGCAG
EscV_R1	TCAGTGGTGGTGTGCTCTGAAATCATTTACCGTTC
EscV_R2	GATCCCCGGGAATTTCAGTGGTGGTGGTGGTGGTGTGCTCT
**pEscT-2HA (pSA10)**	
EscT_F	CAATTTCACACAGGAAACAGATGAATGAGATAATGACGG
EscT_R1	GGTAAGCGTAATCTGGAACATCGTATGGGTACTCATTAATCATGCTCGG
EscT_R2	GATCCCCGGGAATTTCAAGCGTAATCTGGAACATCGTATGGGTAAGCGTAATCTGG
**pEscS**_**K54A**_**-HA (pSA10)**	
K54A_F	TTGCCTTTTTTGCTAGCAATAATAGCAGTGTTTGCT
K54A_R	AGCAAACACTGCTATTATTGCTAGCAAAAAAGGCAA
**pYscS**_**K54A**_	
YK54A_F	CACAGCGATCAATGCGATAACGAAGCCCAGAGTTTGC
YK54A_R	CTTCGTTATCGCA

The TMD1- and TMD2-exchanged *escS* in pSA10 were generated using the template of pEscS_wt_-HA in pSA10. To replace TMD1 of EscS (amino acid positions 21–36) by a TMD backbone sequence of 7-leucine-9-alanine (7L9A), the EscS 37–91 amino acid sequence including the HA tag was amplified using the primer pair EscS_Fc_F/EscS-HA_R2 from the pEscS_wt_-HA vector. The TMD 7L9A backbone was generated by annealing the primer pair 7L9A_F/7L9A_R, by heating the sample to 95°C for 5 min, and then decreasing the temperature to 20°C at a rate of 5°C/min (Tseytin et al., 2018a). The resulting 7L9A backbone was than amplified with the primer pair EscS_7L9A_F/EscS_7L9A_R, to create sequences overlapping the EscS_37−91_ PCR fragment and the sequence upstream of TMD1. EscS_37−91_ was ligated to the 7L9A backbone and amplified using the primer pair EscS_7L9A_F/EscS-HA_R2. Gibson assembly was conducted by amplifying the pEscS_wt_-HA pSA10 vector with the primer pair pSA10_F/EscS_7L9ATM1_R (Table 2), followed by *Dpn*I treatment of the product and subjecting the amplified vector and the EscS_37−90_-7L9A fused PCR fragment to ligation. The resulting construct, pEscS-TMD1_ex_-HA (pSA10), expressed a TMD1-exchanged EscS with an HA tag at its C-terminus. To replace the TMD2 of EscS by a TMD backbone sequence 7L9A, the EscS 1-51 amino acid sequence was amplified using the primer pair EscS-HA_F/EscS_Fn_R (Table 2) from the pEscS_wt_-HA vector. The 7L9A backbone was amplified using the primer pair EscS_TM2_7L9A_F/EscS_TM2_7L9A_R (Table 2). The EscS_1−51_ PCR fragment and the 7L9A backbone were then ligated using overlapping sequences, and amplified using the primer pair EscS-HA _F/EscS_TM2_7L9A_R. Gibson assembly was conducted by amplifying the pEscS_wt_-HA pSA10 vector with the primer pair EscS_7L9AFn_F/ pSA10_R, followed by *Dpn*I treatment of the product and subjecting the amplified vector and the EscS_1−51_-7L9A fused PCR fragment to ligation. The resulting construct, pEscS-TMD2_ex_-HA (pSA10), expressed a TMD2-exchanged EscS with an HA tag at its C-terminus. The *escS-TMD1*_*ex*_*-HA* and *escS-TMD2*_*ex*_*-HA* sequences were then cloned into pET28a(+) by amplifying the TMD-exchanged EscS sequences from pEscS-TMD1_ex_-HA and pEscS-TMD2_ex_-HA (pSA10), using the primer pair EscS-V_5__F/EscS-V_5__R1 and then EscS-V_5__F/EscS-V_5__R2 (Table 2). The pET28a(+) plasmid was amplified with the primer pair pET28_F/pET28_R (Table 2). The PCR products were subjected to digestion by *Dpn*I, purified, and assembled by the Gibson assembly method. The resulting constructs, pEscS-TMD1_ex_-V_5_ and pEscS-TMD2_ex_-V_5_, expressed a TMD1- and TMD2-exchanged EscS, respectively, with a V_5_ tag at their C-terminus in a pET28a(+) vector. All constructs were verified by DNA sequencing.

### Construction of Plasmids Expressing EscT-2HA and EscV-His

To construct EscT-2HA and EscV-His expression vectors, the coding sequences of EscT and EscV were amplified by PCR from EPEC E2348/69 genomic DNA, using the primer pairs EscT_F/EscT_R1 and EscV_F/EscV_R1 (Table 2), respectively, and then with the primer pairs EscT_F/EscT_R2 and EscV_F/EscV_R2 (Table 2), respectively, which fused a 2HA tag to the coding region of *escT* and His tag to the coding region of *escV*. The PCR products were subjected to digestion by *Dpn*I, purified, and assembled by the Gibson assembly method into pSA10 vector that was amplified by PCR using the primer pair pSA10_F/ pSA10_R (Table 2). The resulting plasmids, pEscT-2HA and pEscV-His, expressed EscT protein fused to a C-terminus double HA tag and EscV protein labeled with a C-terminal His tag, respectively. The constructs were verified by DNA sequencing.

### *In vitro* Type III Secretion Assay

T3S assays were performed as previously described (Shaulov et al., 2017; Tseytin et al., 2018a,b). Briefly, EPEC strains were grown overnight in LB supplemented with the appropriate antibiotics, in a shaker at 37°C. The cultures were diluted 1:40 into pre-heated Dulbecco's modified Eagle's medium (DMEM, Biological Industries) supplemented with the appropriate antibiotics, and were grown statically for 6 h in a tissue culture incubator (with 5% CO_2_), to an optical density at 600 nm (OD_600_) of 0.7. To induce protein expression, 0.25 mM IPTG was added to bacterial cultures. The cultures were then centrifuged at 20,000 × *g* for 5 min; the bacterial pellet was dissolved in SDS-PAGE sample buffer and the supernatant, containing secreted proteins, was collected and filtered through a 0.22 μm filter (Millipore). The supernatant was then precipitated with 10% (v/v) trichloroacetic acid (TCA) overnight at 4°C to concentrate proteins secreted into the culture medium. The volume of the supernatants was normalized to the bacterial cultures OD_600_ to ensure equal loading of the samples. The samples were then centrifuged at 18,000 × *g* for 30 min at 4°C, the precipitates of the secreted proteins were dissolved in SDS-PAGE sample buffer, and the residual TCA was neutralized with saturated Tris. Proteins were analyzed on 16% SDS-PAGE gels and stained with Coomassie Blue.

For the *in vitro* secretion assay, *Y. enterocolitica* cultures were inoculated from stationary overnight cultures and incubated at 28°C, for 1.5 h, while shaking. Plasmid-encoded YscS expression was induced by addition of 0.2% arabinose and the *yop* regulon by transferring the culture to 37°C. After 3 h, OD_600_ was measured, cultures were normalized to contain proteins secreted by 0.4 OD units of bacteria, centrifuged at 16,000 × *g* for 2 min to remove the bacteria, and the supernatant samples were precipitated using 10% TCA, overnight at 4°C. Proteins were separated on 12 or 15% SDS-PAGE gels and stained with Coomassie Blue (Expedeon).

### Translocation Activity

Translocation assays were performed as previously described (Baruch et al., 2011). Briefly, HeLa cells (8 × 10^5^ cells per well) were infected for 3 h with EPEC strains that were pre-induced for 3 h for T3SS activity (pre-heated DMEM, statically, in a CO_2_ tissue culture incubator). Cells were then washed with PBS, collected, and lysed with RIPA buffer. Samples were then centrifuged, at maximum speed for 5 min, to remove non-lysed cells and supernatants were collected, mixed with SDS-PAGE sample buffer and subjected to western blot analysis with anti-JNK and anti-actin antibodies (loading control). Uninfected samples and the Δ*escN* mutant strain-infected samples were used as negative controls.

### Immunoblotting

Samples were subjected to SDS-PAGE and transferred to nitrocellulose (pore size: 0.45 μm; Bio-Rad) or PVDF (Mercury, Millipore) membranes. The blots were then blocked for 1 h, with 5% (w/v) skim milk-PBST (0.1% Tween in phosphate buffered saline), incubated with the primary antibody (diluted in 5% skim milk-PBST, for 1 h, at room temperature, unless indicated otherwise), washed and then incubated with the secondary antibody (diluted in 5% skim milk-PBST, for 1 h, at room temperature). Chemiluminescence was detected with the EZ-ECL reagents (Biological Industries). The following primary antibodies were used: mouse anti-HA (Abcam Inc.), diluted 1:1,000; mouse anti-HA.11 (Covance) diluted 1:1,000; mouse anti-V_5_ (Invitrogen), diluted 1:1,000; rabbit anti-MBP (ThermoFisher Scientific), diluted 1:1,000; mouse anti-JNK (BD Pharmingen), diluted 1:1,000 in TBS; mouse anti-DnaK (Abcam, Inc.), diluted 1:5,000; rat anti-intimin (a gift from B. Brett Finlay), diluted 1:2,000; mouse anti-His (Pierce), diluted 1:2,000, mouse anti-actin (MPBio), diluted 1:10,000, and rabbit anti-FLAG (Rockland), diluted 1:2,000. The following secondary antibodies were used: horseradish peroxidase-conjugated (HRP)-goat anti-mouse (Abcam Inc.), HRP-conjugated goat anti-rabbit (Abcam Inc.), and HRP-conjugated goat anti-rat (Jackson ImmunoResearch) antibodies. Representative western blots of at least three independent experiments are presented in the results section.

### Bacterial Fractionation

Bacterial cell fractionation was performed based on a previously described procedure (Gauthier et al., 2003). Briefly, EPEC strains from an overnight culture were sub-cultured 1:50 in 50 mL DMEM, for 6 h, at 37°C, in a CO_2_ tissue culture incubator. Cells were harvested, washed in PBS, and resuspended in 0.25 mL buffer A [50 mM Tris (pH 7.5), 20% (w/v) sucrose, 5 mM EDTA, protease inhibitor cocktail (Roche Applied Science), and lysozyme (100 μg/mL)] and incubated for 15 min, at room temperature, while rotating, to generate spheroplasts. MgCl_2_ was then added to a final concentration of 20 mM, and samples were spun for 10 min at 5,000 × *g*. The supernatants containing the periplasmic fractions were collected. The pellets, which contained the cytoplasm and the membrane fractions, were resuspended in 1 mL lysis buffer (20 mM Tris/HCl pH 7.5, 150 mM NaCl, 3 mM MgCl_2_, 1 mM CaCl_2_, and 2 mM β-mercaptoethanol with protease inhibitors). All subsequent steps were carried out at 4°C. RNase A and DNase I (10 μg/mL) were added and the samples were sonicated (Fisher Scientific, 3 × 15 s). Intact bacteria were removed by centrifugation (2,300 × *g* for 15 min), and the cleared supernatants containing cytoplasmic and membrane proteins were transferred to new tubes. To obtain the cytoplasmic fraction, supernatants were centrifuged (in a Beckman Optima XE-90 Ultracentrifuge with a SW60 Ti rotor) for 30 min at 100,000 × *g*, to pellet the membranes. The supernatants, containing the cytoplasmic fraction, were collected and the pellets, containing the membrane fractions, were washed with lysis buffer and the final pellets were resuspended in 0.1 mL lysis buffer with 0.1% SDS. The protein content of all samples was determined using the Coomassie Plus protein assay (Thermo Scientific) before adding SDS-PAGE sample buffer with β-mercaptoethanol. Intimin, maltose binding protein (MBP), and DnaK were used as markers for the membrane, periplasm and cytoplasm fractions, respectively.

### Chemical Labeling and Blocking of Cysteine Residues

To determine the topology of EscS, EPEC Δ*escS* expressing pEscS_wt_-HA was grown under T3SS-inducing conditions for 6 h, after which chemical labeling and blocking of cysteine residue was performed as previously described, with minor modifications (Gerard et al., 2002). Briefly, bacteria were collected and converted to spheroplasts, as described above. To block the periplasmic thiol groups, spheroplasts were incubated with 1 mM MTSET, at room temperature, for 3 min, and then washed three times with PBS. Blocked and untreated spheroplasts were then incubated with PBS containing 1 mM biotin-HPDP, at room temperature, for 20 min. Subsequently, they were washed three times with PBS and lysed with lysis buffer (150 mM NaCl, 5 mM EDTA, 50 mM Tris [pH 7.5], 1% LDOA). The samples were then centrifuged for 15 min at 10,000 x g and the supernatants were collected and incubated with NeutrAvidin beads (GE Healthcare), overnight, at 4°C, with mild agitation. The beads were then collected by centrifugation and washed three times with lysis buffer, twice with high-salt wash buffer (500 mM NaCl, 5 mM EDTA, 50 mM Tris [pH 7.5], 0.1% LDOA), and once with salt-free wash buffer (50 mM Tris [pH 7.5], 0.1% LDOA). Proteins were eluted by SDS sample buffer containing 200 mM dithiothreitol.

### Co-immunoprecipitation

*Escherichia coli* BL21 (λDE3) transformed with pEscS_wt_-HA or pEscS_wt_-V_5_, were grown to mid-exponential phase in LB and induced with 0.25 mM IPTG (18 h, 16°C). Cells were then harvested by centrifugation (4,000 × *g*, 30 min, 4°C) and washed twice with PBS. The washed pellets were resuspended in lysis buffer, sonicated (Fisher Scientific, 3 × 15 s) and then incubated with 0.1% Nonidet P-40 (NP-40), on ice, for 15 min. Intact cells were removed by centrifugation (18,000 × *g*, 15 min, 4°C). Whole-cell lysates (WCLs) were collected and aliquoted into tubes containing EscS_wt_-HA lysate, EscS_wt_-V_5_ lysate, and a tube that contained both. Samples were brought to similar volumes by adding lysis buffer. Mouse anti-HA antibody (1.5 μg) was then added to all samples and incubated for 30 min, at 4°C. Then, washed protein G slurry beads were added to each sample and incubated on a rotatory wheel overnight at 4°C. Finally, the beads were centrifuged, washed five times with 1 mL lysis buffer, and eluted by adding SDS-PAGE sample buffer and boiling the beads for 10 min. Equal amounts of WCL and eluted fractions were subjected to SDS-PAGE and then western blot analysis with anti-HA and anti-V_5_ antibodies.

To examine protein interactions between EscS and EscR, EscT, EscV, or EscU similar approach was used. Whole cell lysates were made from *E. coli* BL21 (λDE3) transformed with pEscR-3HA, pEscT-2HA, pEscV-His or the non-cleavable EscU mutant, pEscU_N262A_-2HA.

### Crude Membrane Preparation

Bacterial membranes were prepared using the bacterial membrane fractionation protocol described above. Membrane proteins were then extracted by resuspending the membrane pellets in lysis buffer containing 1% n-dodecyl-β-D-maltoside (DDM). The samples were then incubated on a rotary-wheel, for 60 min, at 4°C. Non-solubilized material was removed by centrifuging the samples at 20,000 × *g*, for 15 min, at 4°C. The supernatants were collected and analyzed by Blue-Native PAGE.

### Blue Native-PAGE

Extracted membrane proteins were incubated for 5 min in a blue-native (BN) sample buffer (30% glycerol with 0.05% Coomassie Brilliant Blue G250), and loaded onto a Criterion XT Tris-Acetate 3–8% gradient native gel (Bio-Rad). For electrophoresis, the cathode buffer was 15 mM Bis-Tris and 50 mM Bicine (adjusted to pH 7) and the anode buffer was 50 mM Bis-Tris (adjusted to pH 7). Electrophoresis was carried out on ice, until full separation (5–6 h). The gel was then subjected to western immunoblotting with anti-HA and anti-V_5_ antibodies.

### Site-Directed Mutagenesis

Site-directed mutagenesis of K54A within the EscS-HA (pSA10) construct was performed using the primer pairs K54A_F/K54A_R (Table 2), and within the YscS construct (pBAD) using the primer pairs YK54A_F/YK54A_R (Table 2).

### Fluorescence Microscopy

*Yersinia enterocolitica* were grown under non-secreting conditions, as described above. After 3 h of incubation at 37°C, 1.5 μl of the bacterial culture was mounted on a 1.5% agarose pad in microscopy imaging buffer (100 mM HEPES pH 7.2, 100 mM NaCl, 5 mM ammonium sulfate, 20 mM sodium glutamate, 10 mM MgCl_2_, 5 mM K_2_SO_4_, and 0.5% casamino acids) containing diaminopimelic acid (60 μg/ml) and CaCl_2_ (5 mM) into a depression slide. The samples were visualized in a Deltavision Spectris Optical Sectioning Microscope (Applied Precision), equipped with a UApo N 100x/1.49 oil TIRF UIS2 objective (Olympus), using an Evolve EMCCD Camera (Photometrics). The exposure time was set to 0.2 s, with a light intensity of 32% for bright field or GFP settings. Following image acquisition, images were deconvolved using softWoRx 5.5 (standard “conservative” settings), and further processed with ImageJ-Fiji (National Institute of Health). All images within an experiment were processed using the same settings.

### 3D Model Building

EscR/S/T complex was build based on PDB 6F2D (Kuhlen et al., 2018). For this purpose, amino acid sequences of EscR/S/T were uploaded onto Swiss model server (Bienert et al., 2017; Waterhouse et al., 2018) to generate a 3D model of each monomer. The single monomers were assembled into a 3D complex by overlapping the single chains of 6F2D complex. The generated complex was energy minimized using Swiss PDB viewer (Guex and Peitsch, 1997). Analysis and figures production were done using PyMOL (The PyMOL Molecular Graphics System, Version 2.0 Schrödinger, LLC).

## Results

### 3D Model of EscR_5_S_4_T

**Figure 1:**
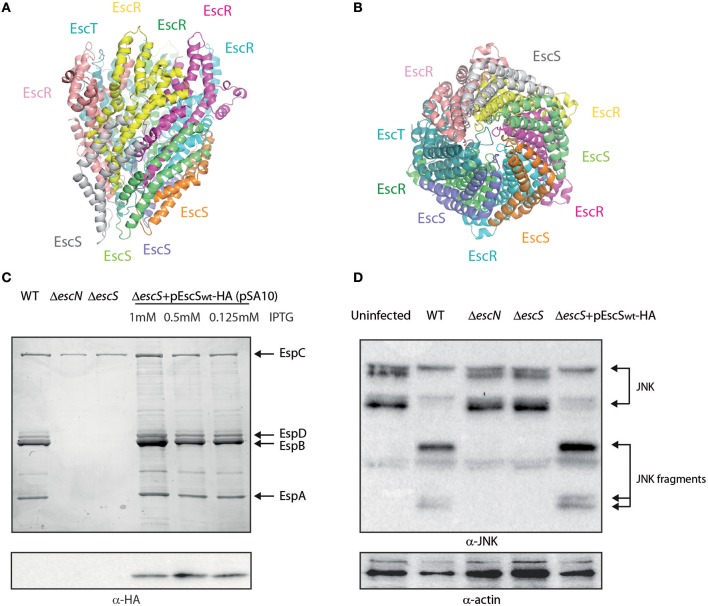
EscS can complement Δ*escS*. **(A)** Side-view of the EscR_5_S_4_T complex based on the PDB 6F2D complex of the flagellar FliP_5_Q_4_R complex of *Salmonella*. Protein subunits are labeled with their corresponding color in the model. **(B)** Bottom-view of the EscR_5_S_4_T complex. **(C)** Protein secretion profiles of EPEC strains grown under T3SS-inducing conditions: wild-type (WT) EPEC, Δ*escN* (a T3SS ATPase mutant), Δ*escS*, and Δ*escS* carrying the pEscS_wt_-HA and treated with different IPTG concentrations. The secreted fractions were concentrated from the supernatants of bacterial cultures and analyzed by SDS-PAGE and Coomassie blue staining. The T3SS-secreted translocators EspA, EspB, and EspD are marked on the right of the gel. Also indicated is the location of EspC, which is not secreted via the T3SS. For the Δ*escN* and Δ*escS* strains, no T3SS activity was observed. The Δ*escS* strain carrying the plasmid encoding EscS_wt_-HA showed proper T3SS activity, regardless of the IPTG concentration used. EscS_wt_-HA expression was detected when bacterial pellets were analyzed on SDS-PAGE and western blot analysis with an anti-HA antibody. **(D)** Proteins extracted from HeLa cells infected with WT, Δ*escN*, Δ*escS*, and Δ*escS* carrying the pEscS_wt_-HA were subjected to western blot analysis using anti-JNK and anti-actin (loading control) antibodies. JNK and its degradation fragments are indicated at the right of the gel. WT EPEC showed massive degradation of JNK relative to the uninfected sample and the samples infected with Δ*escN* or Δ*escS* mutant strains. EPEC Δ*escS* complemented with pEscS_wt_-HA showed similar JNK degradation profile as observed for WT EPEC, indicating a functional complementation.

To evaluate the likelihood that the EscR_5_S_4_T complex adopts similar structure to the FliP_5_Q_4_R of *Salmonella* flagella (PDB 6F2D), the Spa24_5_9_4_29 of *Shigella flexneri* T3SS (PDB 6R6B), and the SpaP_5_Q_4_R of *Salmonella* T3SS (PDB 6PEE) we generated a 3D model using the amino acid sequences of the EscR/S/T proteins (Figures 1A,B). After energy minimized cycles, the backbone RMSD between the FliP_5_Q_4_R and EscR_5_S_4_T complexes was found to be 0.7 Å even though the average sequence identity is 34%. No major collisions were observed in the 3D EscR_5_S_4_T complex, thus suggesting the EPEC T3SS likely adopts similar structure as the other complexes (Kuhlen et al., 2018; Hu et al., 2019; Johnson et al., 2019).

### EscS (SctS) Is Critical for T3SS Activity and a Labeled Protein Can Complement the Null Mutant

It was previously shown that *escS* is crucial for proper T3SS activity and that an *escS* null mutant in the related murine pathogen, *C. rodentium*, is unable to infect host cells or elicit disease in mice (Deng et al., 2004; Tseytin et al., 2018b). To characterize the EscS protein, we fused a hemagglutinin (HA) tag to the C-terminus of EscS and examined whether the labeled protein is functional. For this purpose, we examined whether transformation of pEscS_wt_-HA can complement the T3SS activity of Δ*escS* mutant. T3SS activity is measured by the ability of EPEC strains, grown under T3SS-inducing conditions, to secrete three T3SS translocators (EspA, EspB, and EspD) into the culture supernatant. Indeed, we observed that while WT EPEC demonstrated T3SS activity, the Δ*escS* mutant strain secreted no translocators and displayed a secretion pattern similar to that of the Δ*escN* EPEC strain, deleted for the T3SS ATPase gene (Figure 1C). Complementation of the mutant strain with hemagglutinin (HA)-labeled *escS, in trans*, restored secretion of the translocators, thus suggesting the labeled protein is functional (Figure 1C). Expression of the labeled EscSwt-HA protein was confirmed by SDS-PAGE; EscS_wt_-HA expression was detected in the Δ*escS* mutant strain transformed with pEscS_wt_-HA, regardless of the IPTG-inducing concentrations (Figure 1C). As labeling of SctS proteins of other pathogenic strains was non-trivial and resulted mainly in non-functional proteins or undetectable level of expression (Figure S1), having a functional and detectable EscS protein is of experimental value.

To confirm the functionality of EscS-HA in a bacterial infection model, we examined the ability of EPEC Δ*escS* complemented with pEscS_wt_-HA, to infect HeLa cells and translocate effectors into the host cells. For this purpose, we infected HeLa cells with various EPEC strains (WT, Δ*escN*, Δ*escS*, and Δ*escS* complemented with pEscS_wt_-HA) and examined the cleavage pattern of JNK, a host protein that is cleaved by a translocated EPEC effector, called NleD (Baruch et al., 2011). As expected, WT EPEC induced extensive degradation of JNK, relative to the uninfected sample and to the samples infected with Δ*escN* or Δ*escS* mutant strains (Figure 1D). EPEC Δ*escS* complemented with EscS_wt_-HA showed a JNK degradation profile similar to that observed for WT EPEC, indicating functional complementation by HA-labeled EscS.

### EscS Is Localized to the Bacterial Membrane

**Figure 2:**
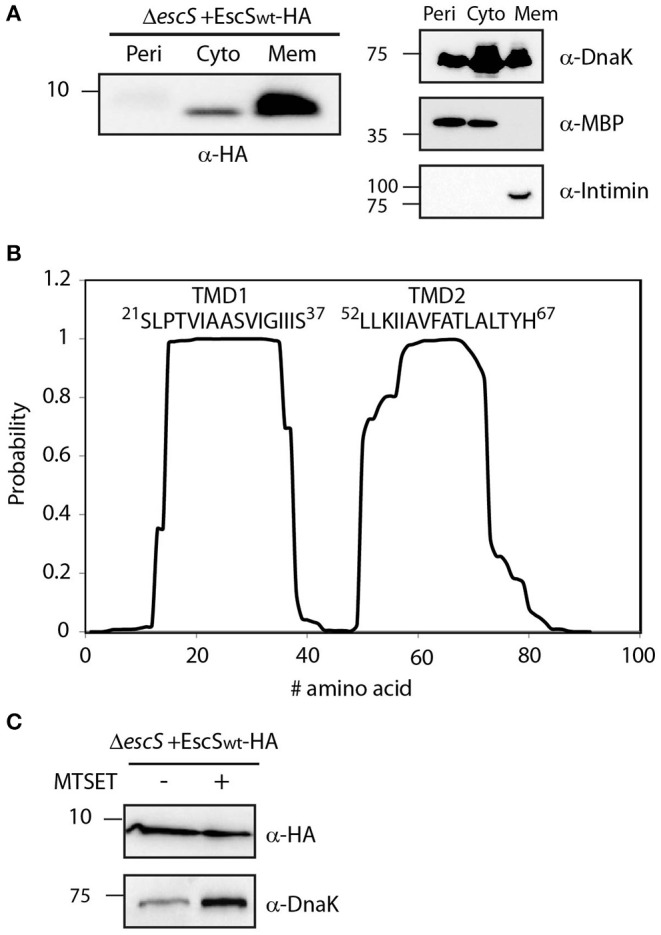
EscS localizes to the membrane fraction and its N-terminal cysteine residue is inaccessible. **(A)** Δ*escS* EPEC carrying the EscS_wt_-HA vector were grown under T3S-inducing conditions and fractionated into periplasmic (Peri), cytoplasmic (Cyto), and membrane (Mem) fractions. The samples were separated on an SDS-PAGE and analyzed by western blotting using anti-HA antibody. To confirm correct bacterial fractionation, the western blots were probed with anti-DnaK (cytoplasmic marker), anti-MBP (periplasmic marker), and anti-intimin (membrane marker) antibodies. **(B)** Analysis of the EscS sequence to rank the probability of each amino acid to be localized within the membrane, using the prediction software TMHMM (Krogh et al., 2001). Two distinct TMDs (TMD1 and TMD2) were identified. The sequences of the core TMDs are presented. **(C)** Spheroplasts of EPEC carrying the EscS_wt_-HA were grown under T3SS-inducing conditions and treated with (+) or without (–) the blocking reagent MTSET. Unblocked cysteine residues were labeled with MTSEA-biotin and biotinylated proteins were recovered with streptavidin-sepharose resin and analyzed by SDS-PAGE and western blot analysis using anti-HA antibody. The samples were also analyzed using an anti-DnaK antibody as a control for inaccessible protein.

EscS, as well as the remaining export apparatus proteins, is predicted to be a membrane protein that is found at the core of the T3SS (Deng et al., 2017; Kuhlen et al., 2018). To determine whether EscS is localized to the membrane, we examined its subcellular localization in EPEC Δ*escS*+pEscS_wt_-HA grown under T3SS-inducing conditions. Whole-cell extracts were fractionated into cytoplasmic, periplasmic and membrane fractions and samples of the fractions were analyzed by western blot analysis with anti-HA antibody. The analysis revealed that EscS_wt_-HA is primarily localized in the membrane fraction (Figure 2A).

To identify the PTMDs of EscS, we analyzed its sequence using TMD prediction software (TMHMM, TMPred, and SACS). Overall, the regions spanning positions 16–37 and 50–70, were ranked to have high probability to adopt a TMD orientation (Figure 2B). To determine the topology of EscS, we exploited the fact that EscS has a single cysteine residue, at position 10, which can be labeled by biotin, through its thiol group. If the cysteine is facing the periplasm, this labeling can be blocked by incubation with the membrane-impermeable reagent, MTSET. We observed that EscS was labeled by membrane-permeable MTSEA-biotin and that pre-treatment of EPEC Δ*escS*+pEscS_wt_-HA spheroplasts with MTSET did not disrupt MTSEA-biotin labeling (Figure 2C). Examination of the biotin labeling of the known cytoplasmic protein, DnaK, revealed that the blocking process had no effect on the labeling of the protein and both treated and untreated samples were labeled with biotin, as expected (Figure 2C). Note that this assay provides qualitative data and therefore the DnaK signal difference detected between the treated and untreated samples is not indicative and probably results from minor technical differences. These results suggest that the N-terminus of EscS is localized in the cytoplasm and if indeed EscS has two PTMDs, it is likely that its C-terminal is also found in the cytoplasm. However, considering the structures of FliP_5_Q_4_R of the *Salmonella* flagellar system, the Spa24_5_9_4_29 of *Shigella flexneri* T3SS, and the SpaP_5_Q_4_R of *Salmonella* T3SS where EscS homologs adopt a hairpin structure, with its N- and C-termini facing the periplasm (Kuhlen et al., 2018; Hu et al., 2019; Johnson et al., 2019) it is likely that the cysteine at position 10 of EscS is buried within the export apparatus complex and therefore may not be accessible for modification by the MTSET reagent.

### Self-Oligomerization of EscS

**Figure 3:**
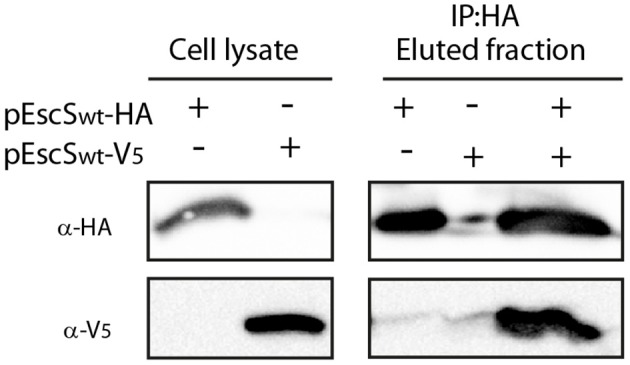
EscS can self-interact. Whole-cell lysates of *E. coli* BL21 (DE3) expressing either EscS_wt_-HA or EscS_wt_-V_5_, were subjected to immunoprecipitation using protein G beads linked to an anti-HA antibody. The lysates were incubated alone or mixed. Whole-cell lysates and elution fractions were separated on a 16% SDS-PAGE and analyzed by western blotting with anti-HA and anti-V_5_ antibodies. EscS-V_5_ was co-eluted with EscS-HA.

It was recently shown that the stoichiometry of the core components of FliP/Q/R is 5:4:1 (Kuhlen et al., 2018; Johnson et al., 2019). This organization suggests that four EscS subunits are present in a single export apparatus complex. To determine whether EscS can self-associate independently of other export apparatus proteins, we expressed HA- and V_5_-labeled EscS in BL21 *E. coli* and examined the *in vitro* interaction between immunoprecipitated EscS subunits. EscS-V_5_ immunoprecipitated together with EscS_wt_-HA, thus suggesting that EscS can self-associate and form at least homo-dimers, *in vitro* (Figure 3). Analysis of whole-cell lysates confirmed the expression of the tagged proteins.

As half of the EscS protein is predicted to be embedded within the membrane and TMDs are known to be involved in protein-protein interactions (Lee et al., 2000; Park et al., 2002; Anbazhagan and Schneider, 2010; Mo et al., 2012; Reuven et al., 2014a), we assessed the involvement of EscS PTMDs in mediating self-interaction. To this end, we utilized the ToxR assembly system, which is designed to detect TMD-TMD self-interactions (Figure S2A). Relatively low levels of self-interaction were observed for both TMD1 and TMD2 of EscS compared to the positive control, glycophorin A (GpA) (Figures S2B–D), suggesting that the self-interaction observed for EscS is likely not mediated by homo-oligomerization of its TMDs.

### EscS Interacts With Additional Export Apparatus Proteins

**Figure 4:**
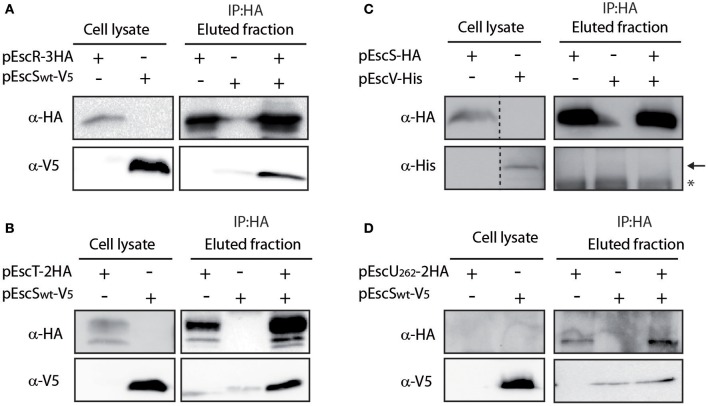
EscS interacts with additional T3SS export apparatus proteins. **(A)** Whole-cell lysates of *E. coli* expressing either EscR-3HA or EscS_wt_-V_5_, were subjected to immunoprecipitation using protein G beads linked to an anti-HA antibody. The lysates were incubated alone or mixed. Samples of whole-cell lysates and elution fractions were loaded on an SDS-PAGE and analyzed by western blotting with anti-HA and anti-V_5_ antibodies. Whole-cell lysates confirmed proper protein expression. EscS-V_5_ co-eluted with EscR, *in-vitro*. **(B–D)** EscT-EscS **(B)**, EscS-EscV **(C)**, and EscU_N262A_-EscS **(D)** immuneprecipitations were examined as in panel A. EscS_wt_-V_5_ co-eluted with EscT-2HA but no interactions were observed between EscS_wt_-HA and EscV-His or between EscS-V_5_ and EscU_N262A_-2HA. In panel C, a black arrow represents the expected size of EscV-His and a star indicates an unspecific signal.

To determine whether EscS interacts with additional export apparatus components, we expressed labeled EscR, EscT, EscV, and EscU proteins and examined their ability to co-immunoprecipitate with EscS, *in vitro*. EscS_wt_-V_5_ co-immunoprecipitated with both EscR-3HA and EscT-2HA (Figures 4A,B), suggesting that EscS can directly interact with the core export apparatus proteins, *in vitro*. Surprisingly, no interaction was detected between EscS and EscV, when EscS_wt_-HA was immunoprecipitated (Figure 4C) or when EscV-His was immunoprecipitated (data not shown), although interaction between the SctV of *Salmonella* flagella (FlhA) and SctS (FliQ) was previously reported (Mcmurry et al., 2004). These results suggest that there are variations between the export apparatus of T3SS and the flagella complex. To examine a possible EscS-EscU interaction, the non-cleavable EscU mutant (N262A) was used; no interaction was detected between EscS and full-length EscU (Figure 4D). Taken together, these findings suggest that EscS interacts directly with EscR and EscT, *in vitro*, while similar interactions were not observed with the EscV or EscU proteins. The inability of EscV and EscU to interact with EscS under these conditions suggests that these interactions are of a weaker affinity or require the presence of additional export apparatus proteins.

### EscS PTMDs Are Crucial for T3SS Activity

**Figure 5:**
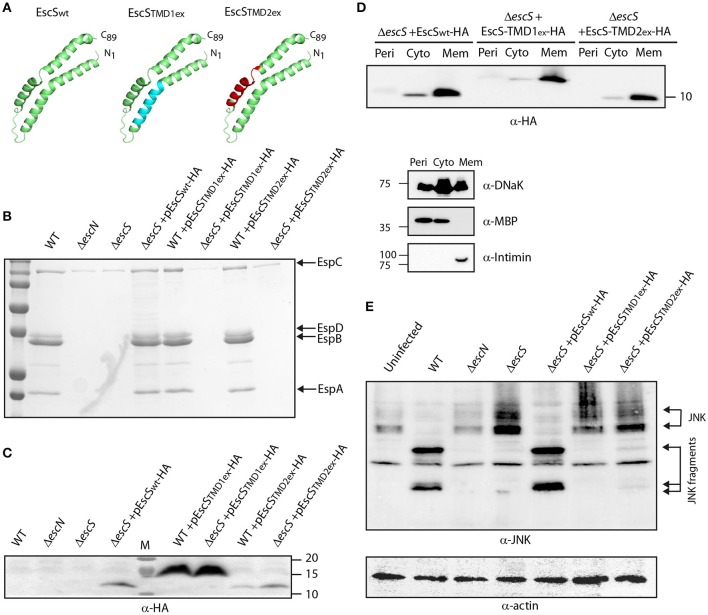
Replacement of EscS TMDs by an alternative hydrophobic sequence abolishes T3SS activity. **(A)** 3D structure of EscS_wt_ monomeric subunit within the EscR_5_S_4_T complex (green). The hydrophobic regions, TMD1_ex_ and TMD2_ex_, that were replaced to 7L9A are marked in cyan and red, respectively. **(B)** Protein secretion profiles of EPEC WT, Δ*escN*, Δ*escS*, and Δ*escS* or WT strains carrying the pEscS_wt_-HA, pEscS-TMD1_ex_-HA or pEscS-TMD2_ex_-HA plasmids grown under T3SS-inducing conditions. The secreted fractions were concentrated and analyzed by SDS-PAGE and Coomassie staining. The secreted translocator EspA, EspB, and EspD are marked on the right of the gel. Also, indicated is the location of EspC, which is not secreted via the T3SS. The Δ*escS* strain carrying the plasmid encoding EscS_wt_-HA showed proper T3SS activity while replacement of either TMD1 or TMD2 to an alternative hydrophobic sequence resulted in non-functional T3SS. Expression of pEscS-TMD1_ex_-HA or pEscS-TMD2_ex_-HA within the WT EPEC strain had no dominant-negative effect on the T3SS activity. **(C)** EscS_wt_-HA, EscS-TMD1_ex_-HA and EscS-TMD2_ex_-HA expression was confirmed in the bacterial pellets obtained in **(B)** by SDS-PAGE and western blot analysis with an anti-HA antibody. EscS-TMD1_ex_-HA migrated slightly slower than EscS_wt_-HA and EscS-TMD2_ex_-HA. **(D)** EPEC Δ*escS* carrying either pEscS_wt_-HA, pEscS-TMD1_ex_-HA, or pEscS-TMD2_ex_-HA, were grown under T3SS-inducing conditions, fractionated into periplasmic (Peri), cytoplasmic (Cyto), and membrane (Mem) fractions and analyzed by western blot analysis with an anti-HA antibody. To confirm correct bacterial fractionation, the western blots were probed with anti-DnaK (cytoplasmic marker), anti-MBP (periplasmic marker), and anti-intimin (membrane marker) antibodies. **(E)** Proteins extracted from of HeLa cells infected with WT, Δ*escN*, Δ*escS*, or Δ*escS* carrying the pEscS_wt_-HA, pEscS-TMD1_ex_-HA or pEscS-TMD2_ex_-HA, were subjected to western blot analysis using anti-JNK antibody and anti-actin (loading control). JNK and its degradation fragments are indicated. WT EPEC showed massive degradation of JNK relative to the uninfected sample and the samples infected with Δ*escN* or Δ*escS* mutant strains. EPEC Δ*escS* complemented with pEscS_wt_-HA showed a JNK degradation profile similar to that of WT EPEC, indicating a functional complementation, while the Δ*escS* transformed with EscS exchanged TMDs (Δ*escS*+ pEscS-TMD1_ex_-HA or Δ*escS*+ pEscS-TMD2_ex_-HA) vectors showed a JNK profile similar to that of the uninfected sample.

EscS is a relatively small protein wherein about half of its sequence is predicted to serve as TMDs at the initial step of EscR_5_S_4_T complex formation and later as hydrophobic helixes at the outer leaflet of the inner-membrane (Hu et al., 2019; Johnson et al., 2019). To determine the role of EscS PTMDs, EscS function was assessed in the absence of either of its PTMDs. Since simple deletion of a PTMD would likely result in altered localization or folding of the protein, which would inevitably alter its activity, we exchanged the 16 central amino acids of each PTMD by a hydrophobic sequence of seven leucines followed by nine alanines (7L9A) (Figure 5A). The 7L9A sequence is known to be sufficiently hydrophobic to support protein integration into the membrane (White and Wimley, 1998, 1999; Sal-Man et al., 2005; Tseytin et al., 2018b). The TMD-exchange constructs (TMD1_ex_ and TMD2_ex_) were transformed into EPEC WT and Δ*escS* strains and their ability to complement the T3SS activity of Δ*escS* mutant strain was assessed. Both pEscS-TMD1_ex_-HA and pEscS-TMD2_ex_-HA failed to complement T3SS in the Δ*escS* strain and had secretion pattern similar to that of the Δ*escN* mutant strain (Figure 5B). These results suggest that the EscS PTMD sequences are critical for the activity of EscS within the T3SS complex and that they do not serve solely as membrane anchors. Interestingly, transformation of EPEC WT with TMD_ex_ EscS proteins had no dominant-negative effect on T3SS activity (Figure 5B). To confirm proper expression of the exchanged-proteins, whole-cell lysates were submitted to western blot analysis using anti-HA antibody. Expression of both EscS WT and TMD_ex_ was detected, however, for an unknown reason EscS-TMD1_ex_-HA showed much higher expression level relative to WT and TMD2_ex_ (Figure 5C). The band corresponding to EscS-TMD1_ex_-HA migrated slightly slower than EscS_wt_-HA and EscS-TMD2_ex_-HA, likely due to altered biophysical properties of the protein, which can directly impact its migration on SDS-PAGE.

To confirm that the exchange of the original PTMDs to the 7L9A sequence had no effect on the localization of the protein, we examined the subcellular localization of EscS-TM1_ex_-HA and EscS-TM2_ex_-HA. Our results showed that EscS-TMD1_ex_-HA and EscS-TMD2_ex_-HA localized mostly to the membrane fraction, similarly to EscS_wt_-HA (Figure 5D). In addition, Δ*escS* transformed with pEscS-TMD1_ex_-HA or pEscS-TMD2_ex_-HA failed to support NleD effector translocation into HeLa cells, and had no impact on JNK degradation (Figure 5E). These results suggest that EscS PTMDs are crucial for full activity of the T3SS complex.

### EscS PTMDs Are Not Involved in EscS Self- and Hetero-Interactions

**Figure 6:**
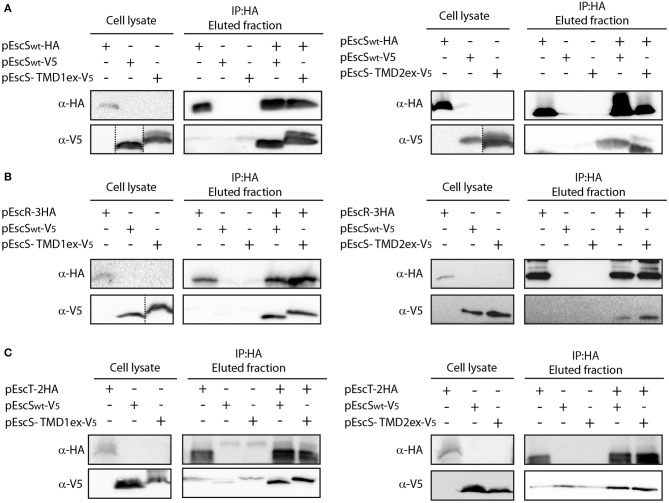
EscS TMDs are not involved in EscS self- and hetero-interactions. Whole-cell lysates of *E. coli* expressing either EscS_wt_-V_5_, EscS-TMD1_ex_-V_5_ or EscS-TMD2_ex_-V_5_ were mixed with EscS_wt_-HA **(A)**, EscR-3HA **(B)**, or EscT-2HA **(C)** and subjected to co-immunoprecipitation using protein G beads linked to an anti-HA antibody. Whole-cell lysates and eluted fractions were subjected to SDS-PAGE and western blot analysis with anti-HA and anti-V_5_ antibodies. EscS self-interaction as well as EscS-EscR and EscS-EscT interactions were not affected by the replacement of the TMD sequences of EscS.

To examine whether the PTMD_ex_ EscS proteins failed to complement the T3SS function due to their involvement in EscS self- or hetero-interactions, whole-cell lysates of *E. coli* BL21 containing EscS_wt_-V_5_, EscS-TMD1_ex_-V_5_, or EscS-TMD2_ex_-V_5_ were mixed with whole cell lysate of EscS_wt_-HA. Immunoprecipitation with anti-HA antibody resulted in co-elution of EscS-TMD1_ex_-V_5_ with EscS_wt_-HA, to levels that were similar to EscS_wt_-V_5_ co-eluted with EscS_wt_-HA (Figure 6A-left panel). Similar results were observed for EscS-TMD2_ex_-V_5_ (Figure 6A-right panel).

To further confirm that EscS PTMDs are not involved in EscS self-interaction, we also examined the self-interaction between EscS-TMD1_ex_ labeled with HA and with V_5_. We found that EscS-TMD1_ex_-V_5_ co-eluted with EscS-TMD1_ex_-HA (Figure S3A) as did EscS-TMD2_ex_-HA with EscS-TMD2_ex_-V_5_ (Figure S3B), further suggesting that the PTMDs of EscS are not required for EscS self-interaction.

To determine whether EscS PTMDs are required for the interaction of EscS with either EscR or EscT, whole-cell lysates of *E. coli* BL21 expressing either EscS-TMD1_ex_-V_5_ or EscS-TMD2_ex_-V5, were prepared and mixed with whole-cell lysate of *E. coli* BL21 expressing EscR-3HA or EscT-2HA. Immunoprecipitation with anti-HA antibody showed that EscS-TMD1_ex_-V_5_ co-eluted with EscR-3HA to similar level as EscS_wt_-V_5_ (Figure 6B-left panel). Similar results were observed for EscS-TMD2_ex_-V_5_, which co-eluted with EscR-3HA (Figure 6B-right panel). In addition, both EscS-TMD1_ex_-V_5_ and EscS-TMD2_ex_-V_5_ co-eluted with EscT-2HA to similar levels as EscS_wt_-V_5_ (Figure 6C). These results suggest that EscS does not interact with EscT or EscR via its PTMDs.

### EscS PTMD2 Is Partially Involved in the Integration of EscS Into the T3SS Complex

**Figure 7:**
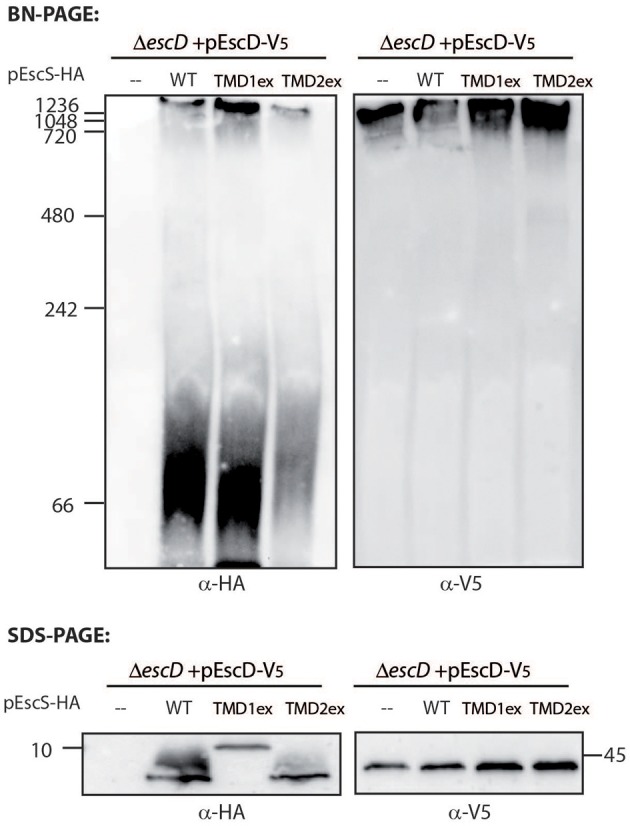
TMD-exchanged versions of EscS associate with the T3SS complex. Membrane protein extracts of Δ*escD* carrying pEscD-V_5_ alone or in combination with pEscS_wt_-HA, pEscS-TMD1_ex_-HA, or pEscS-TMD2_ex_-HA, were incubated in BN sample buffer and then subjected to BN-PAGE (upper panel), SDS-PAGE (lower panel) and western blot analysis using anti-V_5_ and anti-HA antibodies. (**Upper panel**) BN-PAGE analysis showed that while the EscD-V_5_ protein migrated mainly as a large complex at the top of the gel, the EscS WT and the exchanged TMD-versions migrated both as large and small protein complexes. (**Lower panel**) To confirm similar EscS and EscD expression among the samples, membrane protein extracts were analyzed by SDS-PAGE and western blot analysis using anti-HA and anti-V_5_ antibodies. Similar protein expression levels were observed.

To examine whether the PTMD-exchanged EscS variants fail to complement T3SS activity in the Δ*escS* mutant strain due to their inability to properly integrate into the T3SS complex, we prepared crude membranes of Δ*escD* complemented with EscD-V_5_ alone or in combination with either EscS_wt_-HA, EscS-TMD1_ex_-HA, or EscS-TMD2_ex_-HA. EscD forms one of the two concentric inner-membrane rings of the T3SS complex in EPEC (Ogino et al., 2006), and therefore can serve as a marker for the assembled T3SS complex. Crude membranes of the bacterial strains, grown under T3SS-inducing conditions, were prepared and analyzed by a Blue Native (BN)-PAGE and western immunoblotting with anti-HA and anti-V_5_ antibodies. A very large molecular weight, EscD-V_5_-containing complex was detected in all crude membrane samples (Figure 7). A similarly high molecular weight EscS_wt_-HA- or EscS-TMD1_ex_-HA-containing complex was observed in Δ*escD* complemented with EscD-V_5_ together with EscS_wt_-HA or with EscS-TMD1_ex_-HA (Figure 7). However, a much lower signal was observed for the membrane sample of Δ*escD* complemented with EscD-V_5_ and EscS-TMD2_ex_-HA (Figure 7). In addition, a strong HA-positive band was detected above the 66 kDa size marker, which likely indicates an intermediate EscS-containing complex, in the membrane samples of Δ*escD* complemented with EscD-V_5_ together with EscS_wt_-HA or EscS-TMD1_ex_-HA. A similar complex was barely detected in the membrane sample of Δ*escD* complemented with EscD-V_5_ and EscS-TMD2_ex_-HA. To confirm that the altered running pattern of the PTMD2-exchange protein was not due to lower expression of EscS-TMD2_ex_-HA, crude membrane extracts were analyzed by SDS-PAGE and western blotting using both anti-HA and anti-V_5_ antibodies. Similar expression levels were noted for all of EscS variants (Figure 7). Similar complex formation patterns were observed when EscS-TMD2_ex_-HA was expressed in Δ*escS* background; EscS-TMD2_ex_-HA formed much lower levels of high molecular-weight complexes than Δ*escS* complemented either with EscS_wt_-HA or EscS-TMD1_ex_-HA (Figure S4). Overall, these results, suggest that while the exchange of either PTMD1 or PTMD2 with an alternative hydrophobic sequence enabled the formation of high-molecular complexes to some extent, EscS-TMD1_ex_-HA fully preserved the ability to integrate into the T3SS full- or intermediate-complexes, while integration of EscS-TMD2_ex_-HA was impaired.

### A Single Mutation in EscS PTMD2 Abolishes T3SS Activity in EPEC

**Figure 8:**
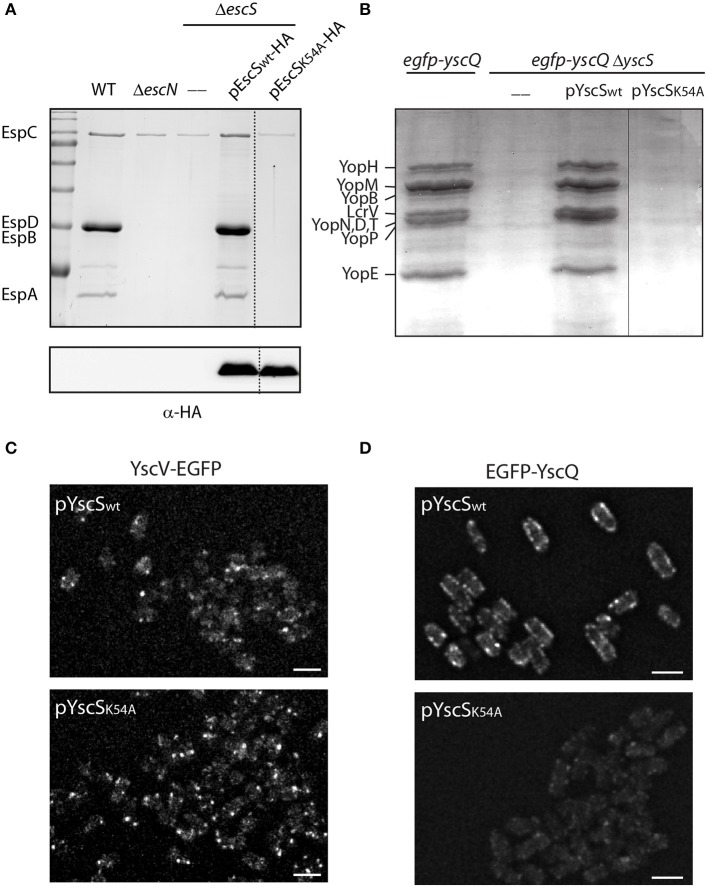
Point mutation at TMD2 disrupts oligomerization of the T3SS cytoplasmic ring. **(A)** Protein secretion profiles of EPEC WT, Δ*escN*, Δ*escS*, and Δ*escS* carrying the pEscS_wt_-HA or pEscS_K54A_-HA grown under T3SS-inducing conditions. The secreted fractions were concentrated from the supernatants of bacterial cultures and analyzed by SDS-PAGE and Coomassie blue staining. The T3SS-secreted translocators EspA, EspB, and EspD are marked on the left of the gel. Also indicated is the location of EspC, which is not secreted via the T3SS. The Δ*escS* strain carrying the plasmid encoding EscS_wt_-HA showed proper T3SS activity, while the plasmid encoding EscS_K54A_-HA was unable to complement T3SS activity. Proper expression of EscS_wt_-HA and EscS_K54A_-HA was detected by analyzing bacterial pellets on SDS-PAGE and western blot analysis with an anti-HA antibody. **(B)** Protein secretion profiles of EGFP-YscQ-expressing WT, Δ*yscS* or Δ*yscS Yersinia* strains carrying pYscS_wt_ or pYscS_K54A_. The secreted fractions were concentrated from the supernatants of bacterial cultures grown under secretion-permissive conditions and analyzed by SDS-PAGE and Coomassie blue staining. Various T3SS-secreted proteins are marked on the left of the gel. **(C,D)** YscV-EGFP **(C)** and EGFP-YscQ **(D)** foci in *Y. enterocolitica* lacking the *yscS* gene and expressing either YscS_wt_ or YscS_K54A_ from a plasmid. All images within an experiment were processed using the same settings. Scale bar: 2 μm.

It has been previously observed that a mutation at position 54 of FliQ, found within the PTMD2 region, abolishes the ability of the bacteria to swarm (Erhardt et al., 2017). In addition, the export apparatus structure of *Salmonella* FliP/Q/R contained an inter-subunit salt bridge between Lys54 and Glu46 of neighboring FliQ subunits (Kuhlen et al., 2018). Protein sequence alignment of EscS with its homologs (FliQ of the *Salmonella* flagella, YscS of the *Yersinia* T3SS, Spa9 of the *Shigella* T3SS, and SpaQ of *Salmonella* SPI-1 T3SS) showed full conservation of the lysine residue at position 54 (Figure S5) and correlated well with the structural alignment we observed (Figure 1A). To examine whether a point mutation at this position disrupts T3SS activity in EPEC, Δ*escS* were transformed with pEscS_K54A_-HA, which encodes a Lys to Ala point mutation at position 54, and grown under T3SS-inducing conditions. The mutant protein failed to complement T3SS activity of a Δe*scS* mutant strain although it was properly expressed (Figure 8A). A similar mutation in the *Yersinia* homolog, YscS, failed to complement Δ*yscS* T3SS activity (Figure 8B). To determine if the mutation at position 54 of the YscS, disrupts the assembly the export apparatus, we examined the formation of fluorescent foci of YscV-EGFP, which was previously shown to occur only upon formation of the export apparatus (Diepold et al., 2011). For this purpose, we grew *Yersinia yscV-egfp* Δ*yscS* mutant strain with a vector that encodes either YscS_wt_ or YscS_K54A_. We observed formation of bright fluorescent foci in both strains, thus suggesting that the mutation in this position does not disrupt the formation of the export apparatus (Figure 8C). Similarly, we observed formation of fluorescent foci of EGFP-YscQ protein within *Yersinia egfp*-*yscQ* Δ*yscS* mutant strain encoding a WT YscS. However, the same strain expressing an YscS_K54A_ protein, exhibited a massive reduction in fluorescence foci formation (Figure 8D), under both secreting and non-secreting conditions (Figure S6). These results suggest that the mutation in lysine 54 impairs T3SS activity, not due to disruption of the export apparatus assembly but likely due to a structural change that prevents proper interaction of the export apparatus with other T3SS sub-complexes, such as the cytoplasmic ring.

## Discussion

The export apparatus is located at the heart of the basal body and was previously suggested to assemble during the initial steps of T3SS formation (Wagner et al., 2010; Dietsche et al., 2016). Among the five proteins that comprise this complex, EscS (SctS) is the smallest component, containing only 81 residues. Nevertheless, EscS, as well as it homologs in other T3SSs, is crucial for T3SS activity and the ability of the bacteria to infect host cells (Deng et al., 2004; Diepold et al., 2011; Yerushalmi et al., 2014; Fabiani et al., 2017; Fukumura et al., 2017; Tseytin et al., 2018b; Wagner et al., 2018). Consensus topology prediction of EscS suggested that the protein adopts a hairpin structure across the inner-membrane, facilitated by its two PTMDs (Taylor et al., 2016). Recent 3D structures of the export apparatus complex of *Salmonella* flagella and *Salmonella* and *Shigella* T3SS confirmed the hair-pin topology of SctS (Kuhlen et al., 2018; Hu et al., 2019; Johnson et al., 2019). However, positioning the complex within the full structures of flagella and T3SS basal bodies, suggested that the export apparatus complex is not embedded within the inner membrane but, rather, resides at the periplasm space, where only the tip of the complex, formed by four SctS monomers, is in contact with the inner-membrane (Kuhlen et al., 2018). Based on these observations, it was suggested that the SctRST complex is initially formed within the inner membrane and during the assembly of the T3SS is extracted out of the inner membrane into the periplasmic space (Kuhlen et al., 2018; Hu et al., 2019; Johnson et al., 2019). Based on this model, the PTMDs of SctRST first function as regular membrane anchors that direct the folding of the protein across the membrane and later may have additional role within the functional T3SS. Using a reductionist approach, where each PTMD was exchanged by an alternative hydrophobic sequence (7L9A), we examined the ability of these mutant proteins to complement the T3SS activity in the Δ*escS* null strain. Both EscS-TMD1_ex_-HA and EscS-TMD2_ex_-HA failed to complement T3SS activity in EPEC Δ*escS* and to translocate effectors into host cells (Figure 5). Although replacement of a TMD with an alternative hydrophobic sequence may seem very destructive and of high likeliness to obliterate the protein function, there are many examples in the literature where TMD replacements resulted in fully functional membrane proteins (Lewis et al., 2001; Li and Blissard, 2008; Huang et al., 2017; Liu et al., 2017; Sastre et al., 2018). To exclude the possibility that the failure to complement T3SS activity by the TMD-exchanged versions was specific for the 7L9A sequence, further examination of additional hydrophobic sequences as well as scrambled sequences is required. Interestingly, expression of EscS-TMD1_ex_-HA or EscS-TMD2_ex_-HA in the WT EPEC background had no dominant-negative effect on the ability of the bacteria to secrete T3SS translocators or to infect host cells (Figure 5). As the PTMD-exchanged versions integrated into the T3SS complexes (Figure 7), at least to some extent, we excluded the possibility that this lack of dominant-negative effect is due to their inability to interact with the complex. As the number of T3SS per EPEC cell is estimated to be 12 (Daniell et al., 2001; Wilson et al., 2001) and each system contains four (Kuhlen et al., 2018), or as recently suggested five (Hu et al., 2019), EscS subunits it is possible that integration of a few TMD-exchanged EscS subunits are not enough to disrupt completely the secretion activity. However, it is possible that such integration alters the kinetics or the regulation of the secretion process. Yet to explore that, more sensitive tools need to be developed.

In attempt to discover what is the functional role of EscS PTMDs within the T3SS complex and why their replacement produced a non-active T3SS, we examined whether the PTMD-exchanged EscSs exhibited impaired interactions compared to the native protein as previously shown for other TMDs (Soetandyo et al., 2010; Fink et al., 2012; Li et al., 2012; Mo et al., 2012; Chavent et al., 2014; Reuven et al., 2014b; Kwon et al., 2015, 2016; Teese and Langosch, 2015). However, our results showed that both PTMD-exchanged versions obtain similar interactions to those seen for the WT protein, suggesting that the PTMDs of EscS are not involved in EscS self-interaction or in its interactions with EscR and EscT.

To determine whether EscS PTMDs contribute to the overall formation/stability of the T3SS complex, solubilized membranes of EPEC grown under T3SS-inducing conditions were analyzed by BN-PAGE. Under these conditions T3SS should preserve its assembled nature and migrate slowly in the gel (Wagner et al., 2010; Dietsche et al., 2016). Assembled T3SS complexes were demonstrated by the presence of EscD, one of the two inner ring-forming proteins of the EPEC T3SS, which was detected in the slowly migrating complexes (> 1 MDa) (Figure 7). While EscS_wt_-HA and EscS-TMD1_ex_-HA migrated in large complexes, which were similar in size to the EscD-containing complexes, as well as in smaller complexes (~70 kDa), EscS-TMD2_ex_-HA showed reduced intensities in both complexes despite similar expression levels for all of EscS variants (Figure 7 and Figure S4). This suggests that the reduced integration of EscS-TMD2_ex_ in the full or intermediate T3SS complexes was due to the involvement of EscS PTMD2 in the interaction between EscS and the other T3SS components. Nevertheless, this observation by itself cannot explain the complete abolishment of the T3SS activity as exchange of PTMD2 was not completely destructive of all T3SS formation.

**Figure 9:**
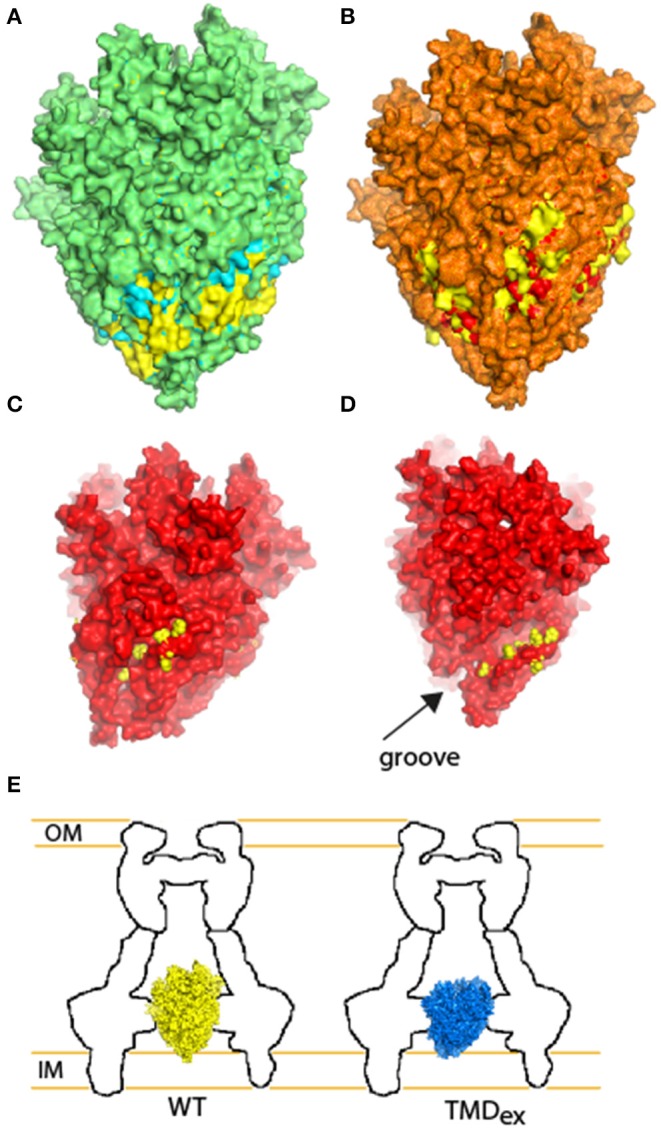
Illustration of the structural adaptations predicted for the TMD-exchanged EscR_5_S_4_T complexes in comparison with the WT complex. **(A)** Overlapping the WT structure of EscR_5_S_4_T (marked in yellow) with the TMD1-exchanged (marked in cyan). Overlaying regions are displayed in green, regions that bulge over the WT complex are marked in cyan (expansion of the TMD1-exchange), and indentations are marked in yellow (concave of the TMD1-exchange). **(B)** Overlapping of WT structure (yellow) and TMD2-exchanged (red). Overlaying regions are displayed in orange, regions that bulge over the WT complex are marked in red, and indentations are marked in yellow. **(C)** Side-view of the TMD2-exchange complex (red) reveals a large void, which is occupied by bulky amino acids in the WT complex (marked in yellow). **(D)** Side-view of the TMD2-exchange complex (red) reveals reduction of density close to a groove found within the EscR_5_S_4_T. The amino acids of the WT complex that are absent from the TMD2 mutant complex are marked in yellow. **(E)** Schematic model of the WT (yellow) and TMD-exchanged (blue) export apparatus complex within the T3SS. Although the complex assembles, the interaction interface between the substructures is altered.

To further examine the involvement of the second PTMD in overall assembly of T3SS, we employed the well-documented fluorescent system of the *Yersinia* T3SS (Diepold et al., 2010, 2011). This system can illustrate the oligomerization of fluorescently labeled central T3SS components by formation of fluorescent foci. Here, we examined the ability of YscV-EGFP, which is one of the export apparatus proteins, and EGFP-YscQ, which is part of the cytoplasmic ring, to form fluorescent foci. While these proteins formed clear foci in WT *Yersinia*, only a few foci were observed when the proteins were expressed in the Δ*yscS* mutant strain (Figure S6). Expression of YscS_wt_ within the Δ*yscS* strain complemented the foci formation of both YscV-EGFP and EGFP-YscQ (Figure 8), while expression of YscS_K54A_ complemented the foci formation of YscV-EGFP foci but not of the EGFP-YscQ (Figure 8). These results, together with the results we obtained for the protein-protein interactions of PTMD-exchanged EscS versions, suggest that the PTMD2 of YscS is not critical for the assembly of the export apparatus *per se* but more likely for orientating its tip toward the cytoplasmic- or the inner-membrane rings. This challenges the common notion that EscS, which is found at the periphery of the minor export apparatus complex (EscR/S/T), mediates the interaction with EscV and EscU. Alternatively, we propose that replacement of the EscS PTMD2 sequence might disrupt the proper orientation of the export apparatus tip, thereby disrupting the ability of the cytoplasmic or inner membrane rings to dock to the T3SS complex and inhibit the T3SS function. To model this hypothesis, we compared structural models of the original EscR_5_S_4_T complex and complexes that contain the PTMD-exchange EscS versions. Overlapping the WT structure with the TMD1-exchanged revealed that replacement of the original PTMD1 resulted in indentation of the lower part of the export apparatus structure (marked in yellow in Figure 9A). Exchange of PTMD2 resulted in a more significant effect and formation of a major void at the center of the EscR_5_S_4_T complex (marked in yellow in Figure 9B); at one interface of the TMD2-exchange complex a large void was formed due to replacement of bulky amino acids (marked in yellow—Figure 9C) while at another interface, we observed a reduction in surface density next to a natural groove found in the complex (Figure 9D). This groove might be occupied by other T3SS proteins or membranous components. Overall, based on the 3D models we speculate that the replacement of EscS PTMDs disorders the interaction interface between the minor export apparatus and additional T3SS substructures or the lipid bilayer (Figure 9E). Since these interactions are complex (each subunit forming interactions with several other subunits), alteration of the interaction interface will likely alter the packing or the orientation of substructures within the complex and therefore prevent proper assembly and function.

## Data Availability Statement

All datasets generated for this study are included in the article/Supplementary Material.

## Author Contributions

IT, BM, ND, KL, AD, and NS-M designed research. IT, BM, ND, and KL performed the research. All authors analyzed the data and contributed to the writing and editing of the manuscript.

### Conflict of Interest

The authors declare that the research was conducted in the absence of any commercial or financial relationships that could be construed as a potential conflict of interest.
