# Supplementary material for: The Role of the Small Export Apparatus Protein, SctS, in the Activity of the Type III Secretion System
Source: Front Microbiol. 2019 Nov 13;10:2551. doi: 10.3389/fmicb.2019.02551 (PMC6863770; doi:10.3389/fmicb.2019.02551)
Supplement: Supplementary file 1 [file Data_Sheet_1.PDF]

Supplementary material

Supplementary materials and methods

Construction of plasmids expressing ToxR-TMD-MBP chimera proteins – Synthetic oligonucleotides pairs encoding a *Nhe*I-*Bam*HI TMD-DNA cassette of 16 core residues of the EscS TMD1 or TMD2 (^{21}SLPTVIAASVIGIIIS^{36}, and ^{52}LLKIIAVFATLALTYH ^{67}, respectively) were phosphorylated, aligned and ligated between the *toxR* transcription activator and the *malE (encodes E. coli* maltose binding protein (MBP)) within a *Nhe*I/*Bam*HI digested ToxR-MBP plasmid. All constructs were verified by DNA sequencing.

Detection of the homo-oligomerization of TMD domains within the membrane – The ToxR transcription activator can be successfully used to assess protein-protein interactions within the *E. coli* membrane. DNA cassettes, encoding single TMDs of EscS (TMD1 and TMD2), *E. coli* aspartate receptor N-terminal TMD (Tar-1), Glycophorin A (GpA) TMD, 16-alanines backbone (A16), 7-leucine-9-alanine backbone (7L9A), and no TMD (ΔTM) were grafted between the cytoplasmic domain of the ToxR transcription activator protein (an oligomerization-dependent transcriptional activator) and the periplasmic moiety of the maltose binding protein (MBP). The presence of the MBP moiety directs the localization of the chimera protein to the periplasm and, therefore, assists the TMD to become embedded within the inner-membrane. In the assay, the ToxR-TMD-MBP plasmids, containing different TMDs, were transformed into *E. coli* FHK12 cells, which contain a reporter gene, β-galactosidase, under the control of the *ctx* promoter. Oligomerization of the investigated TMD results in association of the ToxR transcription activator, which only then becomes active and can bind the *ctx* promoter to initiate transcription of a downstream reporter gene, *lacZ*. Quantification of oligomerization is performed by calculating the activity of β-galactosidase, namely, by measuring the levels of a yellow color (OD_{405}) associated with the cleavage product of the β-galactosidase substrate, *o*-nitrophenylgalactose (ONPG). Monitoring the activity of β-galactosidase for 20 min, at intervals of 30 sec, yields the V_{max} of the reaction and is presented as Miller units when normalized to the original cell content (measured at OD_{600}). We used the GpA TMD sequence as a positive control for strong homo-oligomerization, The N-terminal TMD of the *E. coli* aspartate receptor (Tar-1) as a reference for moderate oligomerization (Sal-Man et al., 2004), and the A16 and 7L9 sequences as controls for non-oligomerizing sequences

Maltose complementation assay – Membrane insertion and correct orientation of the chimera proteins were examined as described previously. Briefly, PD28 cells (a *malE*-deficient *E. coli* strain) transformed with the different ToxR-TMD-MBP plasmids were cultured overnight. The overnight cultures were washed twice with PBS and were used to inoculate an M9 minimal medium supplemented with 0.4% maltose and chloramphenicol.

Bacterial growth was measured at different time points by a spectrophotometer at 600 nm. Since PD28 cells are unable to grow on minimal medium containing maltose as the only carbon source, only cells that expressed the chimera protein in the correct orientation, where the TMD is embedded within the inner membrane and the MBP facing the periplasm, were able to utilize maltose and allow cell growth. A construct with a deleted TMD (ΔTMD) served as a negative control, since the chimera protein was expected to reside in the cytoplasm and, therefore, was unable to compensate for the *malE* deficiency.

Larkin, M.A., Blackshields, G., Brown, N.P., Chenna, R., Mcgettigan, P.A., Mcwilliam, H., Valentin, F., Wallace, I.M., Wilm, A., Lopez, R., Thompson, J.D., Gibson, T.J., and Higgins, D.G. (2007). Clustal W and Clustal X version 2.0. *Bioinformatics* 23**,** 2947-2948.

Sal-Man, N., Gerber, D., and Shai, Y. (2004). The composition rather than position of polar residues (QxxS) drives aspartate receptor transmembrane domain dimerization *in vivo*. *Biochemistry* 43**,** 2309-2313.

Supplementary Figures

Figure S1

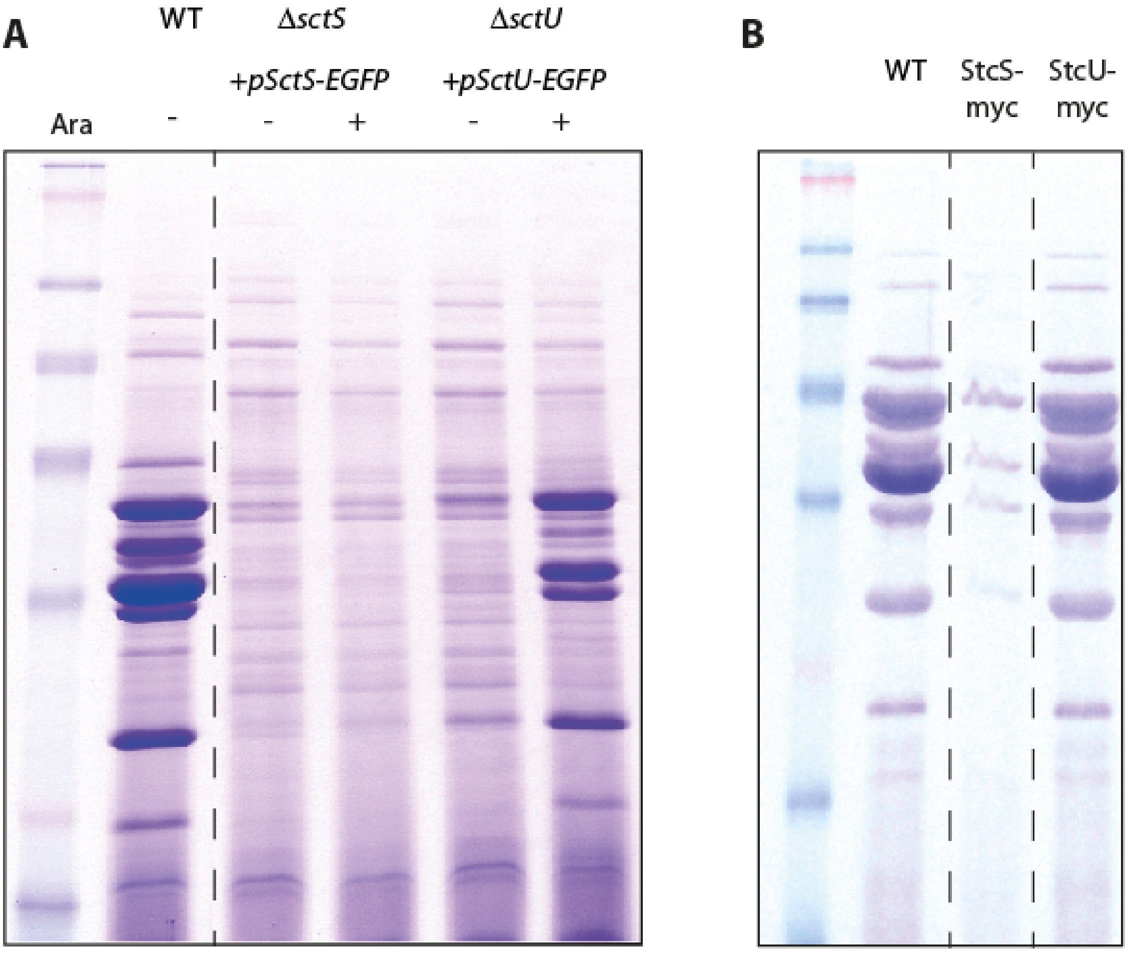

S1 Fig. **Labeled SctS cannot complement T3S in *Yersinia enterocolitica*.** (A) Protein secretion profiles of WT *Yersinia* and mutant strains complemented with C-terminal EGFP fusion of the corresponding gene in-trans. The bacteria were grown in the presence or the absence of 0.15% arabinose (Ara) under secretion-permissive conditions. The secreted fractions from $3x10^9$ bacteria were concentrated and analyzed by SDS-PAGE and Coomassie blue staining. While expression of SctU-EGFP complemented T3S activity, the expression of SctS-EGFP did not. (B) Protein secretion profiles of myc-labeled genes replacing the wild-type genes by allelic exchange. The secreted fractions were analyzed as panel A. While labeled SctU can preserve T3S activity labeled SctS cannot.

Figure S2

A

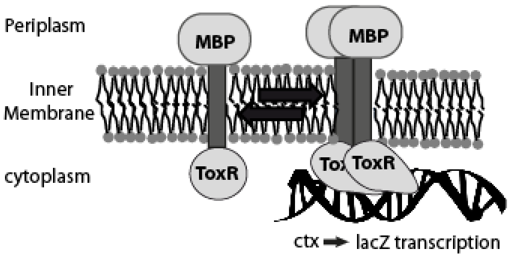

Construct name	Inserted TMD sequence
GpA	183 **ASLIIFGVMAGVIGT**GILI 200
A16	183 AS**AAAAAAAAAAAAAAAA**GILI 200
7L9A	183 AS**LLLLLLLAAAAAAAAA**GILI 200
EscS TMD1	183 AS**SLPTVIAASVIGIIIS**GILI 200
EscS TMD2	183 AS**LLKIIAVFATLALTYH**GILI 200

B

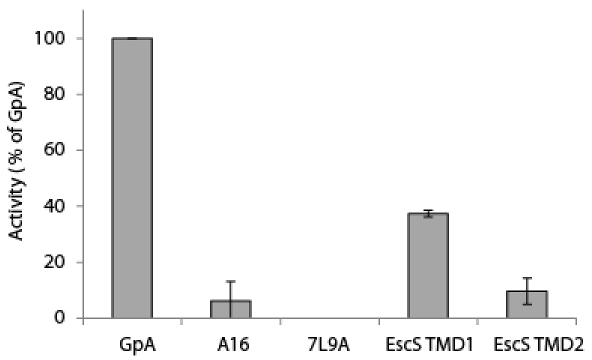

C

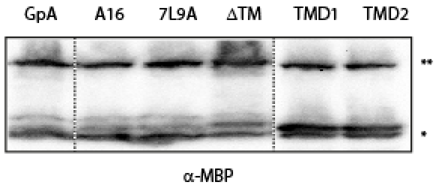

D

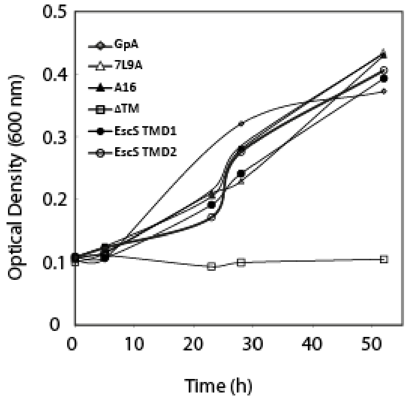

S2 Fig. **EscS TMD oligomerization activity. (A) Schematic illustration of a ToxR assembly system.** Oligomerization of the TMDs promotes the activation of the transcription activator ToxR, which binds the *ctx* promoter and initiates *lacZ* transcription. The TMD sequences that were inserted between the ToxR transcription activator and the maltose binding protein in the ToxR-TMD-MBP plasmid are presented. **(B)** The LacZ activities of FHK12 bacterial strains expressing the ToxR-TMD-MBP chimeras. The activities of well characterized dimerizing (GpA) and non-dimerizing (A16 and 7L9A) TMDs are also shown. The oligomerization ability of EscS TMD1 was low to moderate and of EscS TMD2 was low compared to the activity of GpA TMD. Bars represent the standard deviation of at least three independent experiments. **(C)** Whole-cell lysates of FHK12 expressing the ToxR-TMD-MBP chimera protein with different TMD sequences, were analyzed on a 12% SDS-PAGE and by immunoblotting using an anti-MBP antibody. The ToxR-TMD-MBP chimera protein (65 kDa) is marked with (**) and the endogenous MBP (40 kDa) is marked with (*). **(D)** Correct membrane integration of the ToxR-TMD-MBP chimera proteins was tested by assessing their ability to functionally complement *malE* deficiency in the PD28 bacterial strain. PD28 bacteria were transformed with a

plasmid encoding a chimera protein containing the EscS TMD1 (•), TMD2 (○), GpA (◇), Tar-1 (■), 7L9A (△), A16 (▲), or in the absence of a TMD (ΔTM, □), and were grown in a minimal medium containing maltose. All bacterial cultures showed similar growth curves, indicating proper membrane integration. As expected, the ΔTM negative control showed no growth.

Figure S3

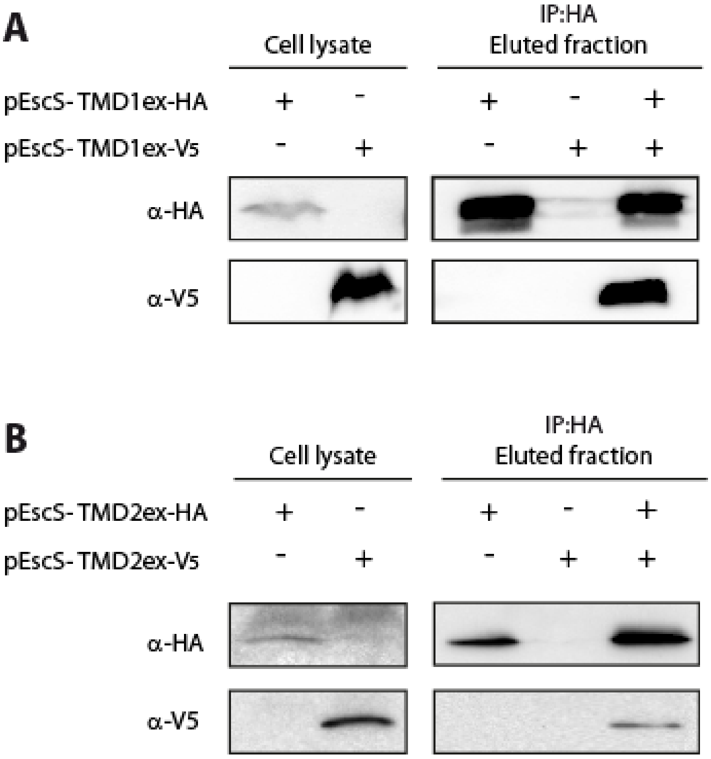

S3 Fig. **TMD-exchanged EscS variants can self-interact. (A)** Whole-cell lysates of *E. coli* BL21 (DE3) expressing either EscS-TMD1$_{ex}$-HA or EscS-TMD1$_{ex}$-V$_5$ were subjected to immunoprecipitation using protein G beads linked to an anti-HA antibody. The lysates were incubated alone or mixed. Samples of whole-cell lysates and elution fractions were loaded on a 16% SDS-PAGE and analyzed using western blot analysis with anti-HA and anti-V$_5$ antibodies. EscS-TMD1$_{ex}$-V$_5$ co-immunoprecipitated with EscS-TMD1$_{ex}$-HA, *in vitro*. **(B)** EscS-TMD2$_{ex}$-V$_5$ co-immunoprecipitated with EscS-TMD2$_{ex}$-HA, *in vitro*, using the protocol described in panel A.

Figure S4

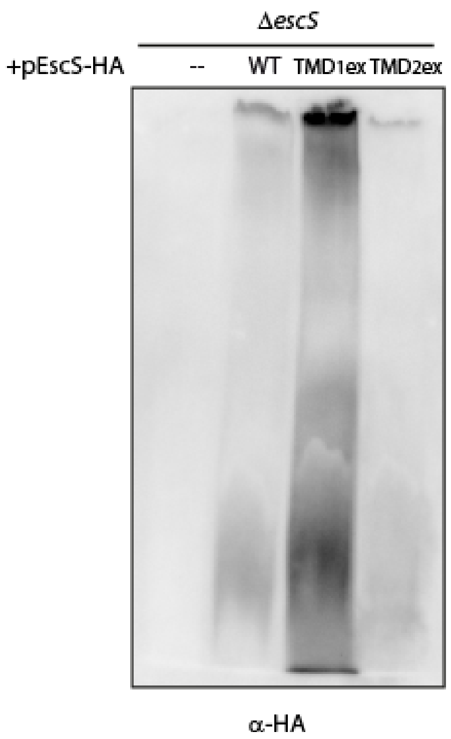

S4 Fig. **EscS localization in T3SS complexes**. Membrane protein extracts of Δ*escS* alone or carrying $pEscS_{wt}$-HA, pEscS-$TMD1_{ex}$-HA, or pEscS-$TMD2_{ex}$-HA, were incubated in BN sample buffer and then subjected to BN-PAGE and western blot analysis using an anti-HA antibody. BN-PAGE analysis showed a lower level of high molecular weight complex formation for Δ*escS* expressing EscS-$TMD2_{ex}$-HA as compared to Δ*escS* expressing either $EscS_{wt}$-HA or EscS-$TMD1_{ex}$-HA.

Figure S5

```
tr|B7UMC0|B7UMC  MDTGYFVQLCVQTFWIIFILSLPTVIAASVIGIIISLVQAITQLQDQTLPFLLKIIAVFATLA
sp|P0A1L5|FLIQ   MTPESVMMMGTEAMKVALALAAPLLLVALITGLIISILQAATQINEMTLSFIPKIVAVFIAII
sp|P69982|YSCS   MSQGDIIHFTSQALWLVLVLSMPPVLVAAVVGTLVSLVQALTQIQEQTLGFVIKLIAVVVTLF
tr|Q6XVW3|Q6XVW  MS--DIVYMGNKALYLILIFSLWPVGIATVIGLTIGLLQTVTQLQEQTLPFGIKLIGVSISLL
tr|A0A1R3D2B9|A  MD--DLVFAGNKALYLVLILSGWPTIVATIIGLLVGLFQTVTQLQEQTLPFGIKLLGVCLCLF
cons             *    .:    ::: : : ::      * : *  :.:.*: **::: ** *  *::.*   :

tr|B7UMC0|B7UMC   LTYHWMGTTIINFSSIIFEMIPKVNG
sp|P0A1L5|FLIQ    VAGPWMLNLLLDYVRTLFSNLPYIIG
sp|P69982|YSCS    ATASWLGNELHSFAEMTMMKIQGI-R
tr|Q6XVW3|Q6XVW   LLSGWYGEVLLSFCHEIMFLIKSG-V
tr|A0A1R3D2B9|A   LLSGWYGEVLLSYGRQVIFLALAK-G
cons                  *    : .:    :
```

S5 Fig. **Sequence alignment of the SctS export apparatus protein.** A standard protein BLAST alignment is presented by ClustalW (Larkin et al., 2007) for EscS of the *E. coli* T3SS (B7UMC0), FliQ of *Salmonella* flagella (P0A1L5), YscS of the *Yersinia* T3SS (P69982), Spa9 of the *Shigella* T3SS (Q6XVW3), and SpaQ of the *Salmonella* T3SS (A0A1R3D2B9). A high level of conservation was observed within the loop region found between the TMD sequences, marked in bold letters, including 100% conservation of the lysine residue at position 54 of EscS of EPEC T3SS.

Figure S6

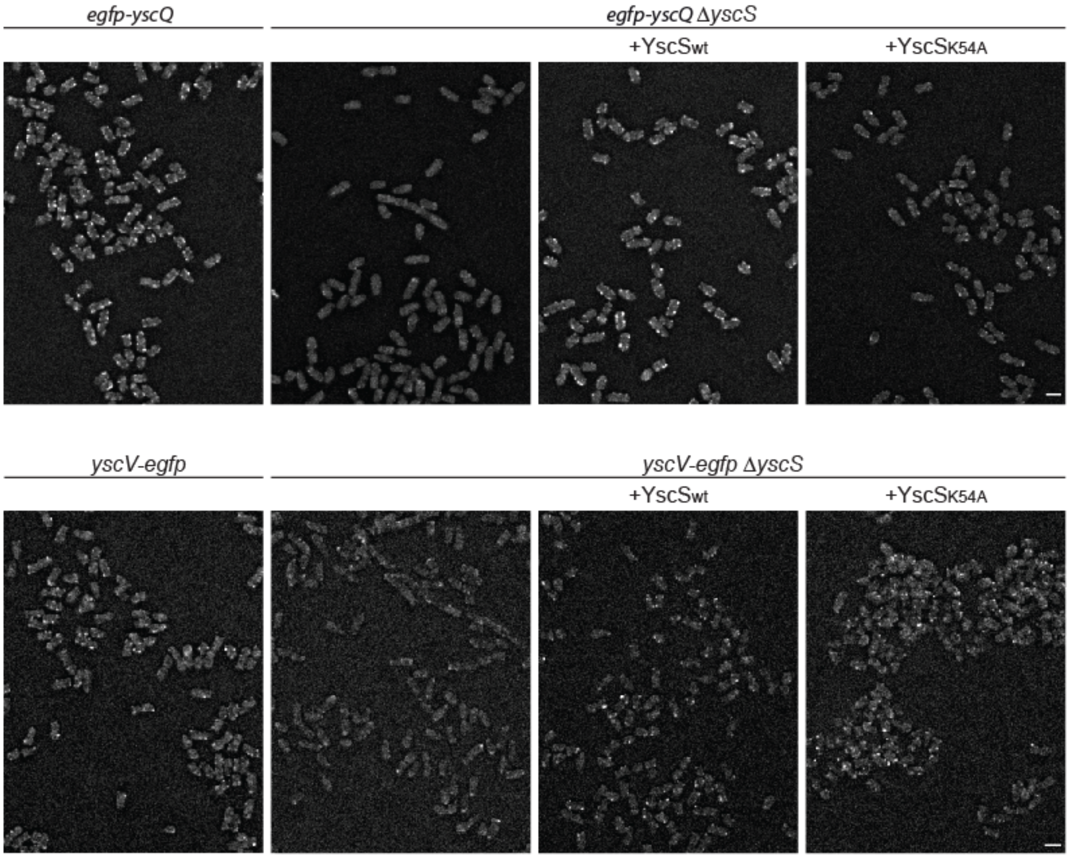

S6 Fig. **Point mutation in YscS TMD2 disrupts oligomerization of the cytoplasmic ring.** EGFP-YscQ (upper panel) and YscV-EGFP (lower panel) foci in *Y. enterocolitica* (WT and Δ*yscS* strains) grown under non-secretion conditions. Transformation of $YscS_{wt}$ resulted in complementation of the foci formation, while transformation of $YscS_{K54A}$ was able to complement the foci formation in cells expressing YscV-EGFP but not in those expressing EGFP-YscQ. Scale bar: 2 μm.

Full-size blots corresponding to the images in the manuscript (unrelated samples are covered by white boxes):

Figure 1:

C.

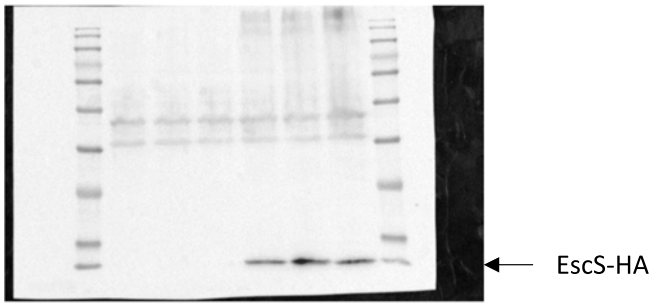

← EscS-HA

α-HA

D.

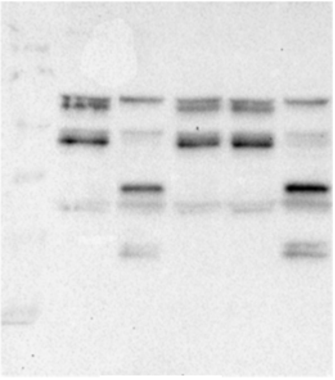

α-JNK

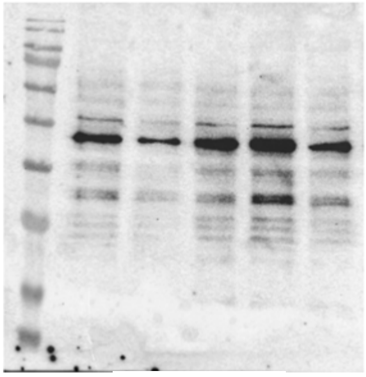

α-actin

Figure 2:

A.

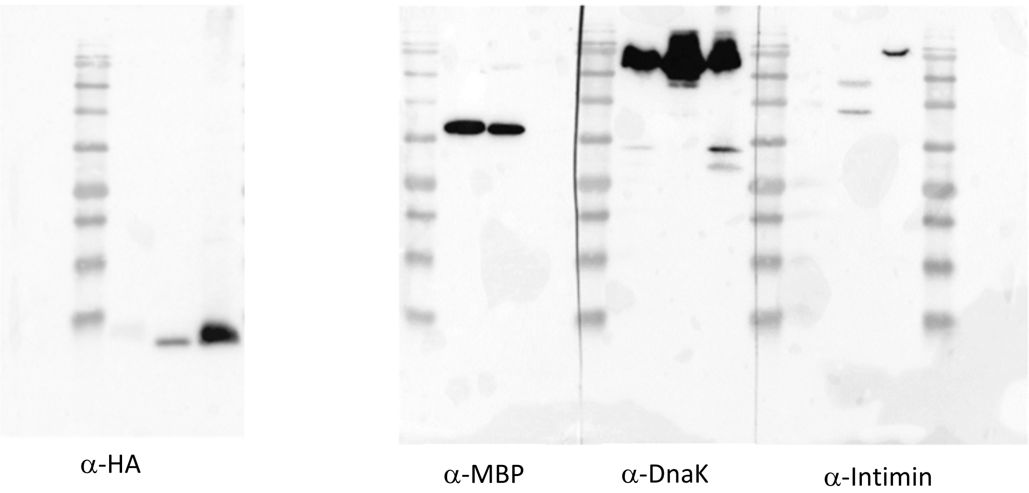

C.

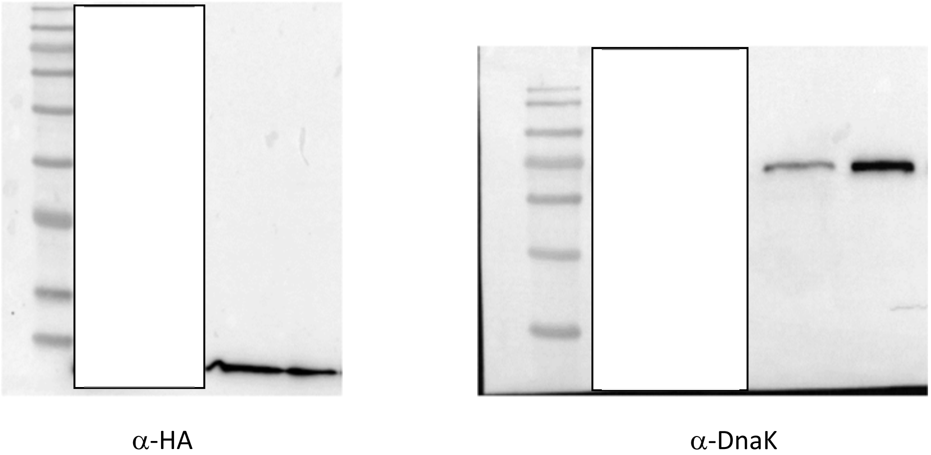

Figure 3:

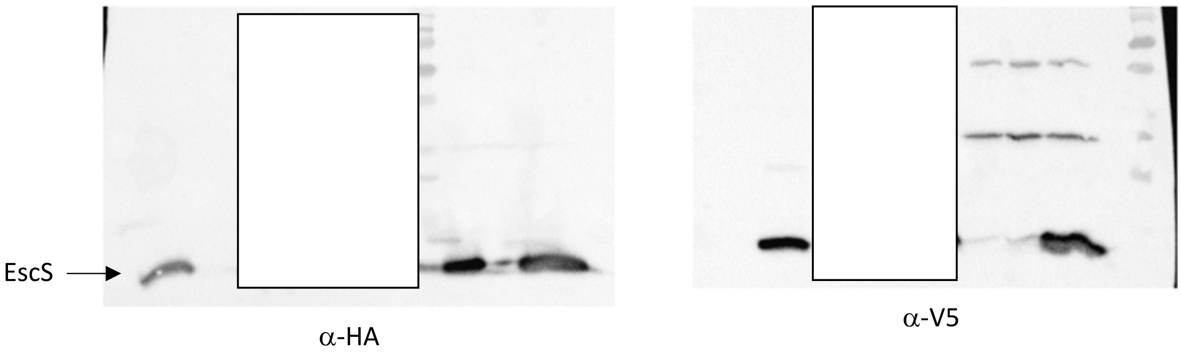

Figure 4:

A.

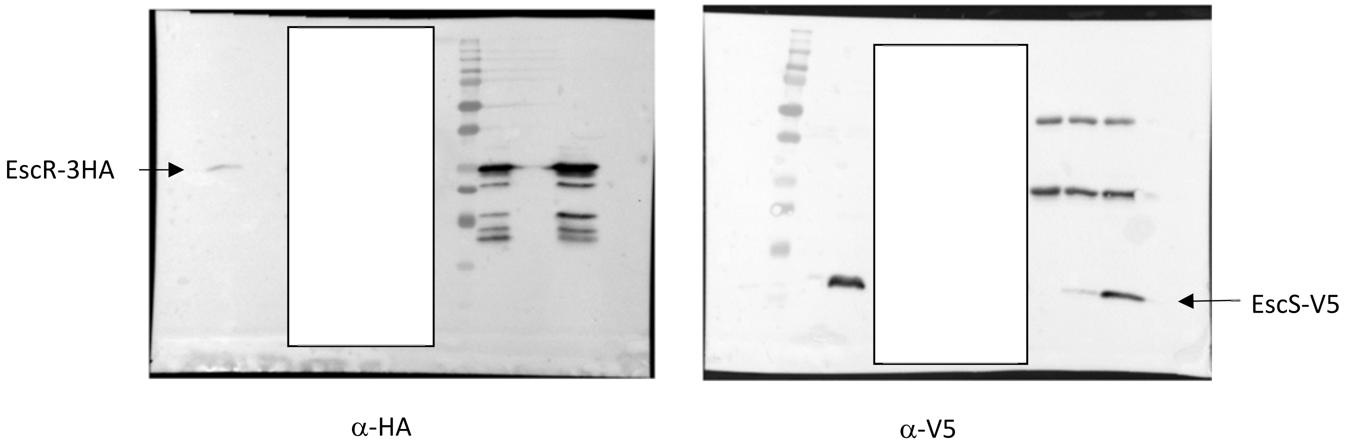

B. Upper part of the membrane was blot with anti-HA antibody and the lower part with anti-V5.

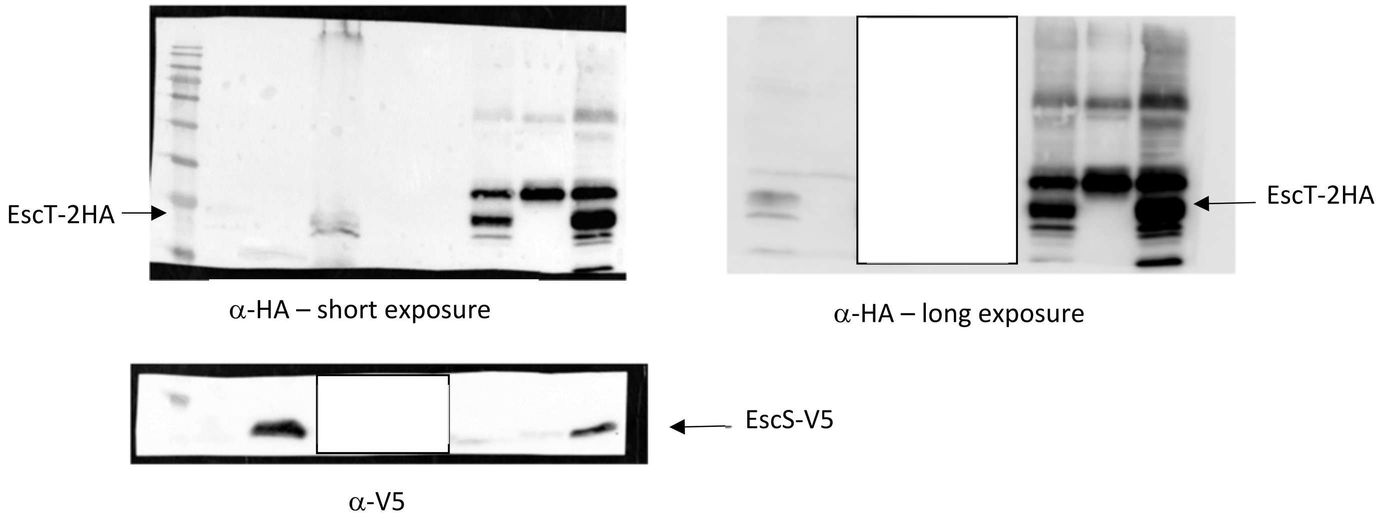

C. Upper part of the membrane was blot with anti-His antibody and the lower part with anti-HA.

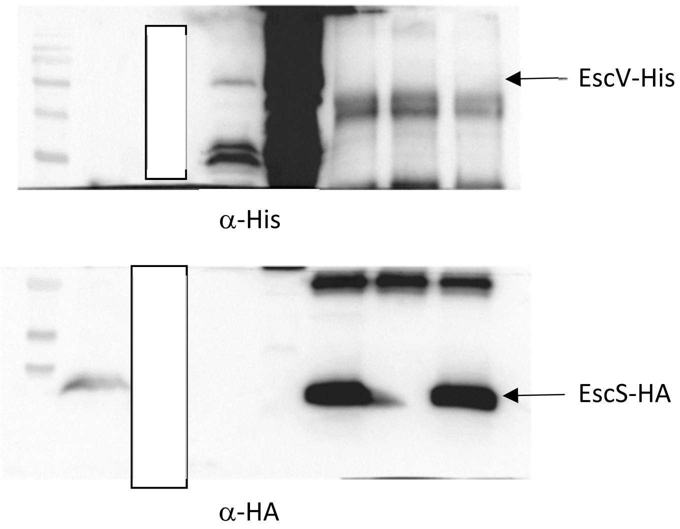

D.

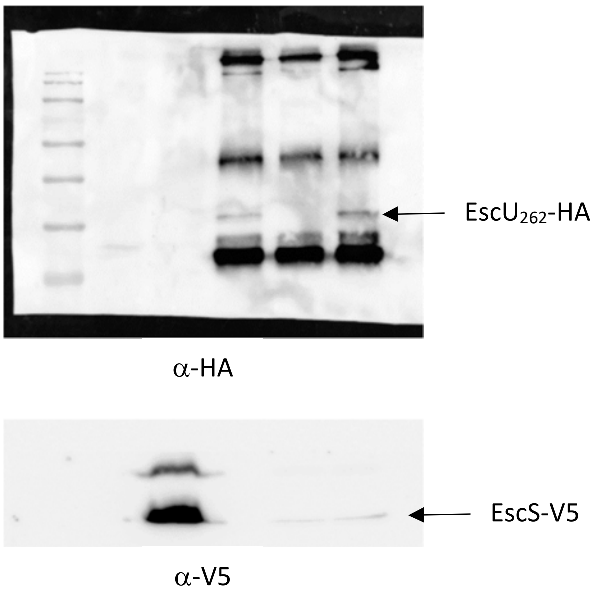

Figure 5:

C.

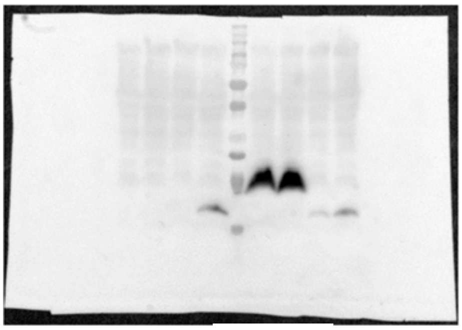

α-HA

D.

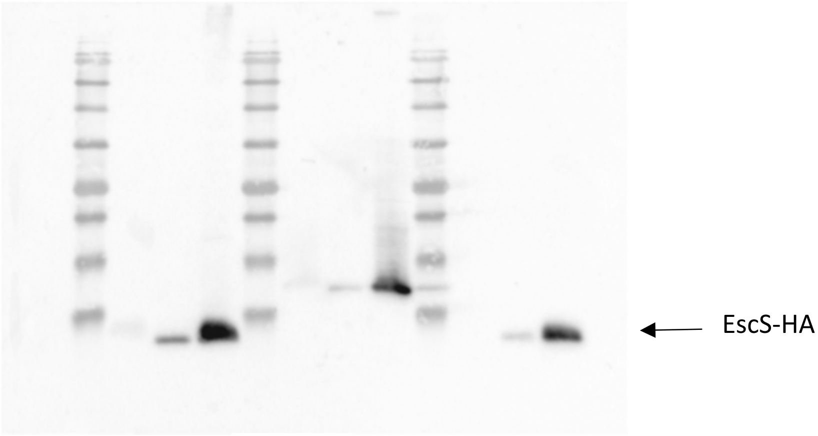

α-HA

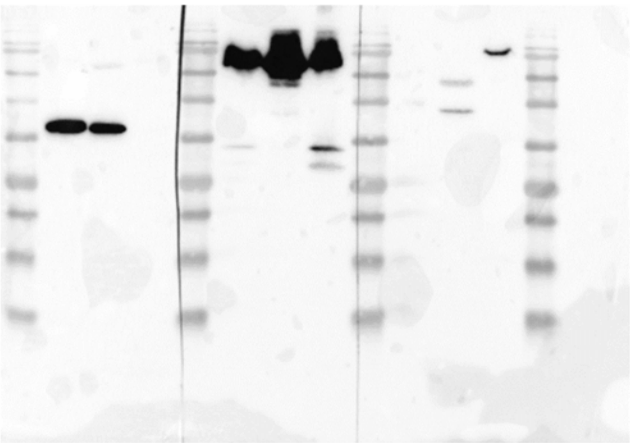

α-MBP α-DnaK α-Intimin

E.

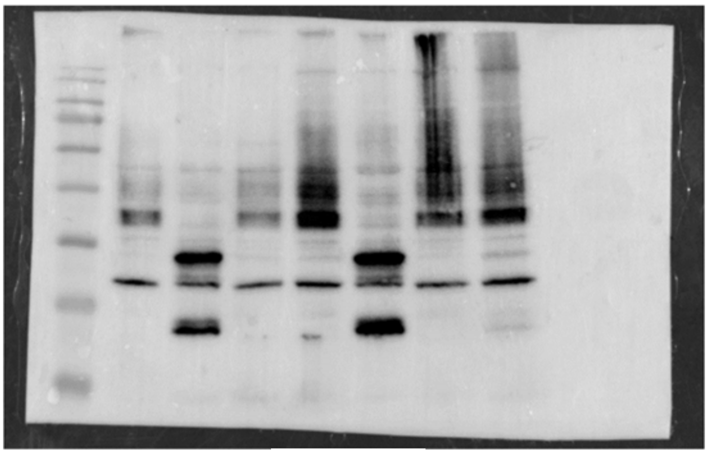

α-JNK

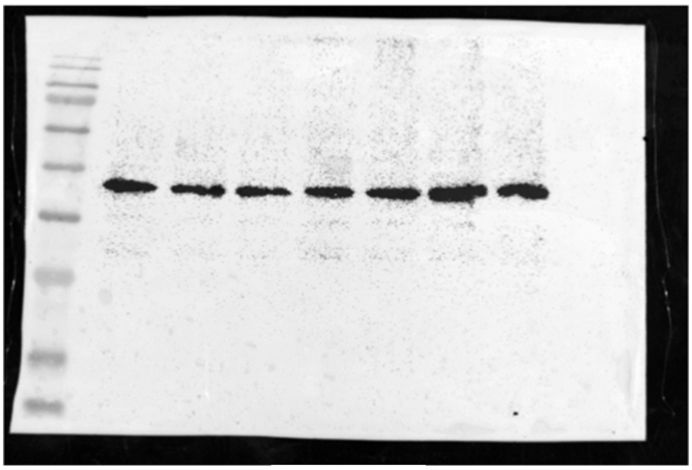

α-actin

Figure 6:

A.

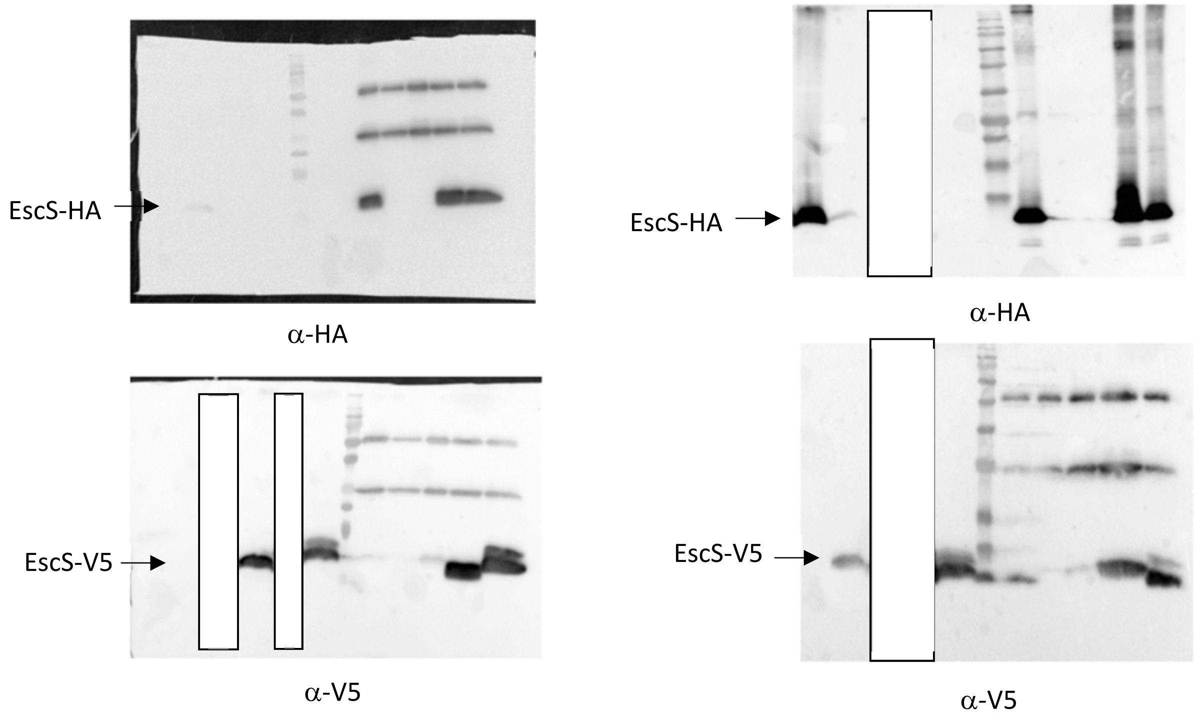
EscS-HA
α-HA
EscS-HA
α-HA
EscS-V5
α-V5
EscS-V5
α-V5

B.

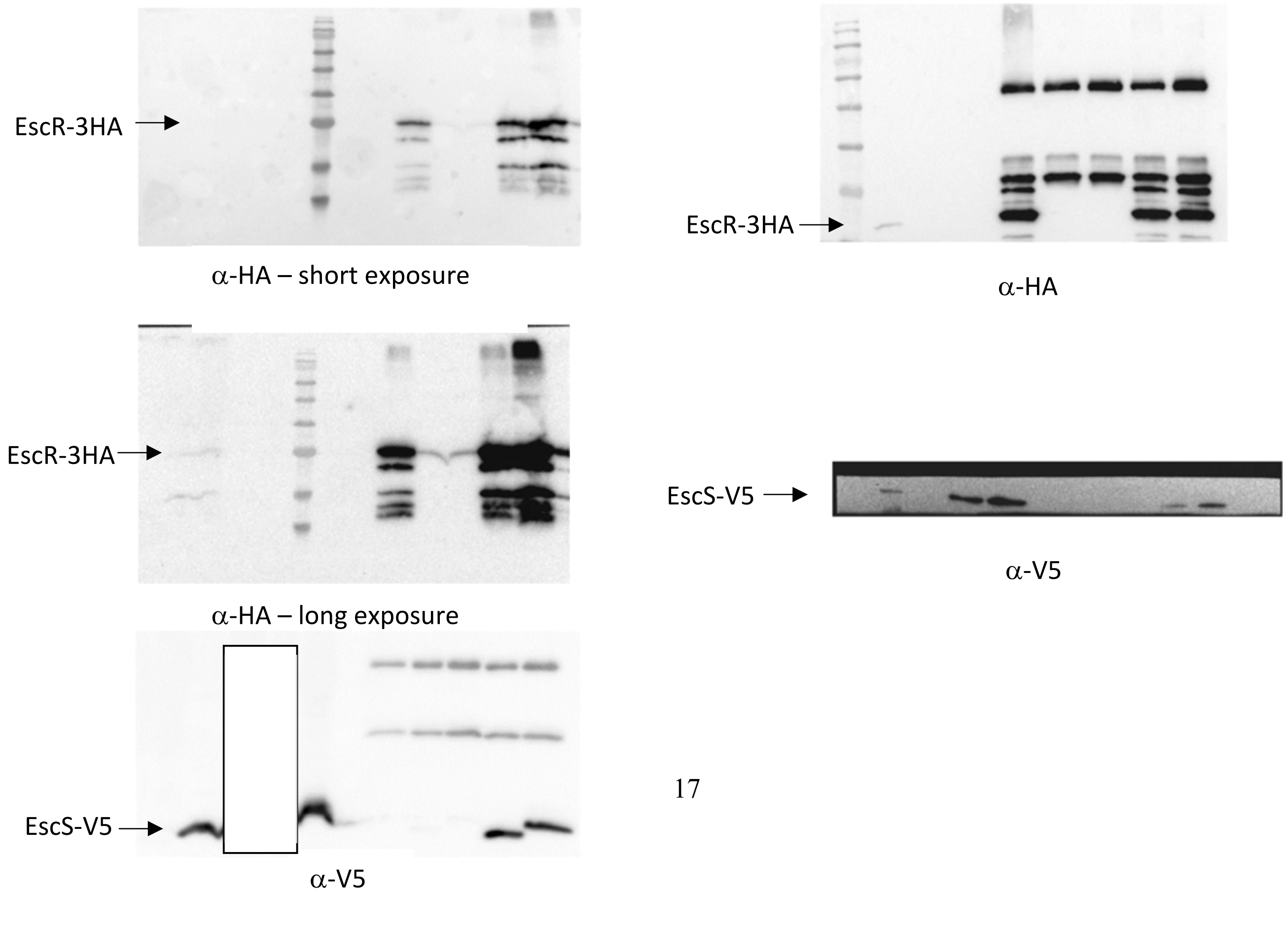
EscR-3HA
α-HA – short exposure
EscR-3HA
α-HA
EscR-3HA
α-HA – long exposure
EscS-V5
α-V5
EscS-V5
α-V5

C.

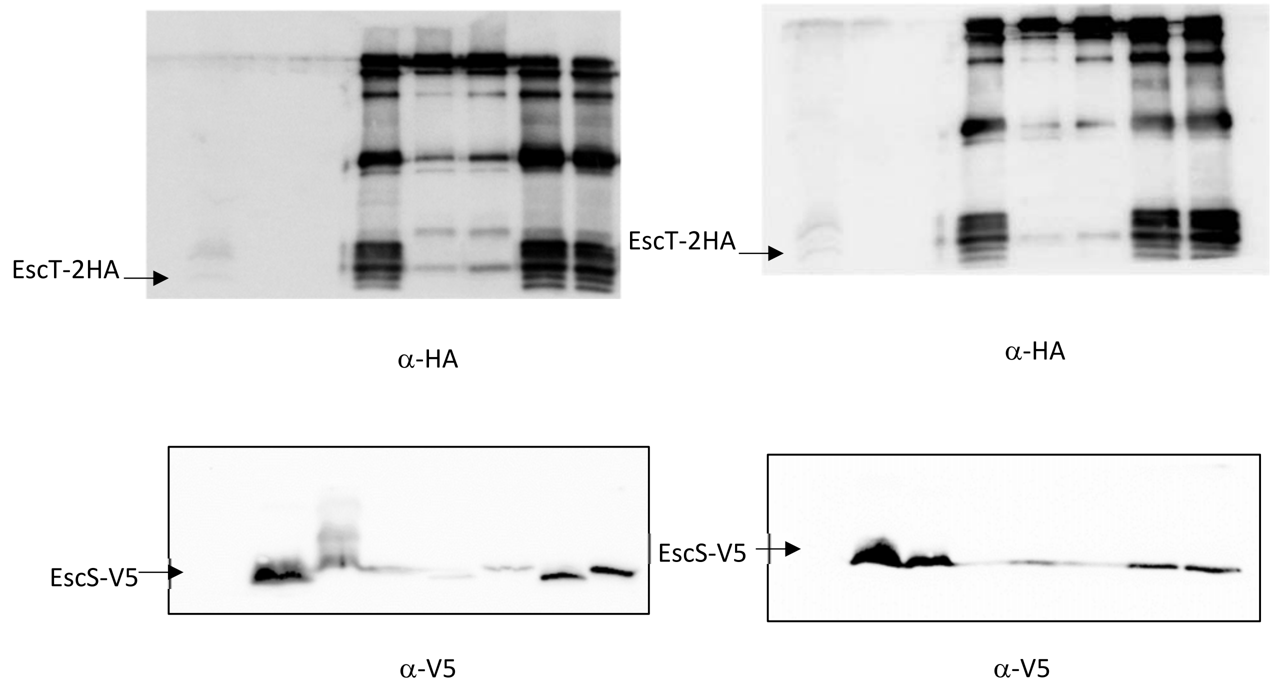

Figure 7:

BN-PAGE:

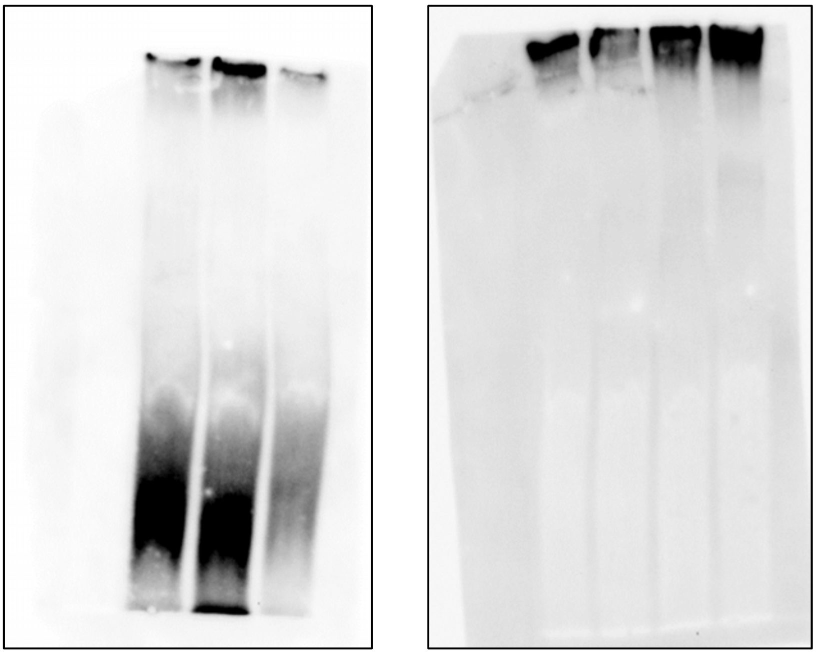

α-HA α-V5

SDS-PAGE: The membrane was cut between the 25 and 35 kDa markers; the upper part was blotted using anti-V5 antibody and the lower part with anti-HA antibody.

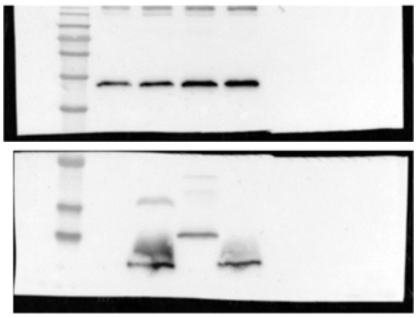

α-V5

α-HA

Figure 8:

A.

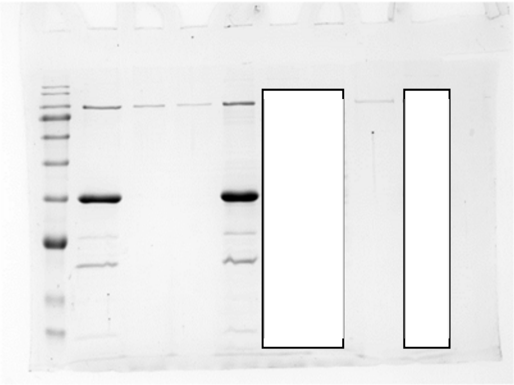

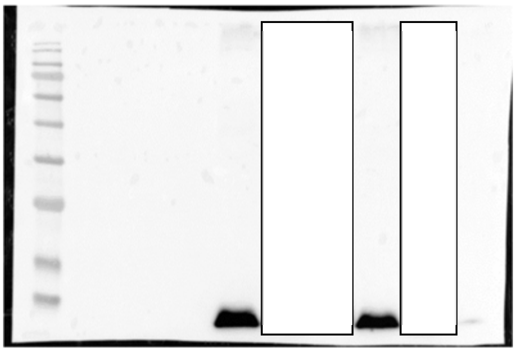

α-HA
